# Magnetizing Biotech–Advances in (In Vivo) Magnetic Enzyme Immobilization

**DOI:** 10.1002/elsc.70000

**Published:** 2025-03-13

**Authors:** Gizem Ölçücü, Karl‐Erich Jaeger, Ulrich Krauss

**Affiliations:** ^1^ Institute of Bio‐ and Geosciences IBG‐1: Biotechnology Forschungszentrum Jülich GmbH Jülich Germany; ^2^ Institute of Molecular Enzyme Technology Heinrich Heine University Düsseldorf, Forschungszentrum Jülich GmbH Jülich Germany; ^3^ Department of Biochemistry University of Bayreuth Bayreuth Germany

**Keywords:** biocatalysis, in vivo enzyme immobilization, magnetic nanoparticles, magnetic protein aggregates, magnetosomes

## Abstract

Industrial biocatalysis, a multibillion dollar industry, relies on the selectivity and efficacy of enzymes for efficient chemical transformations. However, enzymes, evolutionary adapted to mild biological conditions, often struggle in industrial processes that require harsh reaction conditions, resulting in reduced stability and activity. Enzyme immobilization, which addresses challenges such as enzyme reuse and stability, has therefore become a vital strategy for improving enzyme use in industrial applications. Traditional immobilization techniques rely on the confinement or display of enzymes within/on organic or inorganic supports, while recent advances in synthetic biology have led to the development of solely biological in vivo immobilization methods that streamline enzyme production and immobilization. These methods offer added benefits in terms of sustainability and cost efficiency. In addition, the development and use of multifunctional materials, such as magnetic (nano)materials for enzyme immobilization, has enabled improved separation and purification processes. The combination of both “worlds,” opens up new avenues in both (industrial) biocatalysis, fundamental science, and biomedicine. Therefore, in this review, we provide an overview of established and recently emerging methods for the generation of magnetic protein immobilizates, placing a special focus on in vivo immobilization solutions.

**Abbreviations**
BG: β‐glucosidase
CatIBs: catalytically‐active inclusion bodies
CatMPAs: catalytically‐active magnetic protein aggregates
CLEAs: cross‐linked enzyme aggregates
CLECs: cross‐linked enzyme crystals
Coh: cohesin
CP: capsid protein
Doc: dockerin
FKBP: FK506 binding protein
FRB: rapamycin‐binding domain of mTOR
IBs: inclusion bodies
LLPS: liquid–liquid phase separation
MBs: magnetic beads
mCLEAs: magnetic cross‐linked enzyme aggregates
MNPs: magnetic nanoparticles
MPAs: magnetic protein aggregates
OPD: organophosphohydrolase
PAD: phenolic acid decarboxylase
PHA: polyhydroxyalkanoate
POI: protein of interest
RBD: receptor binding domain
TRAbs: TSHR antibodies
TrkA: tropomyosin receptor kinase A
TSH: thyroid stimulating hormone
TSHR: thyroid stimulating hormone receptor
VLPs: virus‐like particles

## 1 Introduction

The use of microbes, and therefore indirectly enzymes, for chemical transformations is as old as human civilization. Since around 10,000 AD, humankind, although for most of that time unknowingly, has been using microbial fermentation to produce alcoholic beverages like beer and wine, as well as food products like bread, cheese, and yogurt [1, 2, 3, 4]. Today, industrial biocatalysis is a multibillion‐dollar industry [5], taking advantage of the high selectivity of enzymes often allowing chemical transformations with high chemo‐, regio‐, and stereoselectivity [6, 7]. Moreover, by definition, enzymes as biologically produced macromolecules are a renewable, biodegradable resource [8, 9], whose industrial use can contribute to the sustainable management of resources, especially, if they are produced by growing the respective production host strains (which are often bacteria or yeasts) on inexpensive renewable resources. While enzymes as biological catalysts are evolutionary adapted to mild reaction conditions and an aqueous environment, industrial processes often demand harsh conditions, including elevated temperatures, extreme pH values, and the presence of organic solvents [10, 11, 12]. Such conditions, however, destabilize enzymes, resulting in rapid inactivation and thus essentially reduced product yields. Therefore, in light of sustainability and the commercially efficient use of enzymes, it is vital to obtain enzyme preparations that are highly stable, easy to handle and reuse, increasing space–time yields while decreasing the environmental burden associated with their production. Enzyme immobilization, which involves confining one or more enzymes in a defined space, was originally developed in the mid‐20th century to primarily address the challenges associated with enzyme recovery and reuse [13, 14, 15, 16, 17]. However, it often also leads to their stabilization [18], making enzyme immobilization an important strategy that can promote the industrial use of enzymes. The conventional approach to immobilizing enzymes entails confining them within or attaching them to inorganic or organic carrier materials. Over time, a plethora of materials and immobilization strategies have been devised to achieve this goal [13]. Alternatively, enzymes can be immobilized by rendering them insoluble in water, for example, by crystallization/aggregation followed by cross‐linking, yielding cross‐linked enzyme crystals (CLECs) and cross‐linked enzyme aggregates (CLEAs) [15, 19].

In the last years, in the wake of the synthetic biology revolution, more and more solely biological, in vivo immobilization methods have been developed that enable the simultaneous production and immobilization of enzymes in one step, that is, during heterologous overproduction of the target enzyme [9]. In terms of both sustainability and costs, the use of in vivo ‐generated immobilizates is advantageous, since their production does not require additional working steps (e.g., cross‐linking or adsorption) or, often chemically derived carrier materials, whose production can amount to approximately 50% of the costs associated with enzyme immobilization [20]. Typically, target enzymes are engineered by fusing them with other protein modules. This fusion either facilitates the self‐assembly of the recombinant fusion proteins into nano‐ or micrometer‐sized supramolecular structures within (microbial) cells or enables the attachment to co‐produced biogenic scaffolding/carrier materials [9]. Another trend seen in recent years is the use of immobilization materials possessing additional functions, such as, for example, being magnetic, which enables magnetic separation, purification [21], and easy mixing inside bioreactors [22], or even stimulus responsiveness to control catalyst function by light, temperature, or magnetic fields [23, 24, 25, 26]. In particular, enzyme immobilization within/on magnetic nanomaterials and magnetic beads (MBs) has seen increased application recently [21, 27, 28]. While the use of MBs and magnetic nanomaterials has matured beyond an obscure academic discipline, with applications found in biomedicine, environmental remediation (including waste water treatment) [29], and oil/energy industries [21], the use of magnetic enzyme immobilizates in synthetic chemistry still represents an emerging field. Similarly, as also outlined above, synthetic biology approaches have recently enabled the development of solely biologically produced magnetic enzyme immobilizates [30, 31, 32, 33, 34, 35, 36, 37].

With the present review, we, therefore, strive to provide an overview of conventional strategies for enzyme immobilization with focus on magnetic (nano)materials, explore recent in vivo solutions for the generation of magnetic enzyme immobilizates, highlight emerging application fields in biotechnology and biomedicine, and suggest future research avenues based on the state of the art.

## 2 Enzyme Immobilization Methods

**FIGURE 1:**
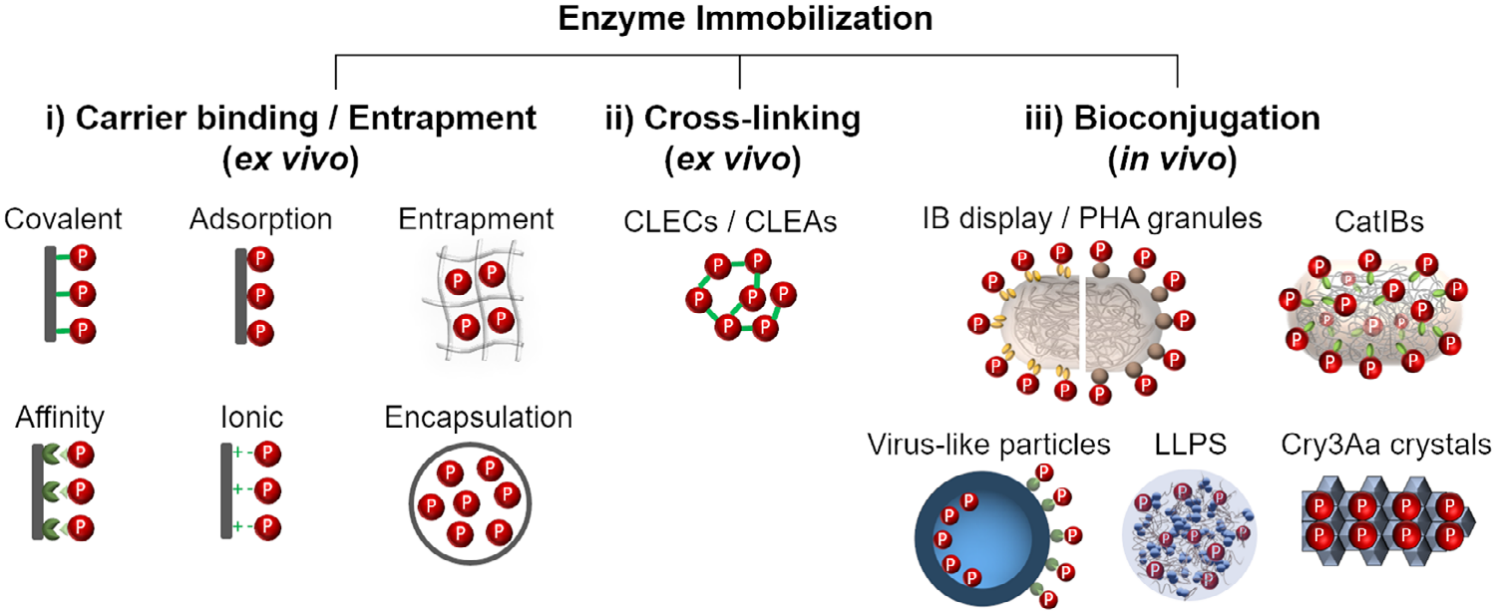
Strategies used for enzyme immobilization. Enzyme immobilization methods can be divided in ex vivo and in vivo methods, with ex vivo methods including (i) methods in which the protein of interest (POI, shown as “P” in the figure) is carrier bound/entrapped in carrier materials and (ii) ex vivo generated immobilizates that do not require external carriers, for example, CLECs/CLEAs. In contrast, (iii) bioconjugation‐based in vivo methods do not rely on carrier materials. Representations for the depicted in vivo methods are adapted from [9]. CatIBs, catalytically‐active inclusion bodies; CLEAs, cross‐linked enzyme aggregates; CLECs, cross‐linked enzyme crystals; IB, inclusion body; LLPS, liquid–liquid phase separated protein condensates PHA, polyhydroxyalkanoate; POI, protein of interest.

As mentioned earlier, there are numerous methods for enzyme immobilization, which can be classified into the following three categories when the use of carrier materials and the in vivo/ex vivo nature of the methods are considered: (i) ex vivo methods, which rely on the production and release of the protein of interest (POI) from the cells, followed by the attachment/confinement of the POI within/on suitable carrier materials; (ii) ex vivo methods that rely on crosslinking of the POI, where the target enzymes are released from the cells and upon crosslinking serve as the carrier themselves; and finally, (iii) fully in vivo methods that do not use carriers but instead rely on bioconjugation, where the production and immobilization of the POI take place as a single step inside the cells (Figure 1). Proportional to the range of available immobilization methods, there are also a plethora of excellent resources and reviews [38, 39, 40, 41, 42] that describe them in detail with different foci; those that focus on classical and recently developed, novel carrier materials [43, 44, 45, 46, 47, 48], industrial applications [39, 49, 50, 51], as well as those that focus on the comparatively more recent, immobilization methods [9, 52–55]. Therefore, the methods depicted in Figure 1 will only be briefly outlined here.

### 2.1 Carrier‐Dependent, Ex Vivo *Vivo* Methods for Enzyme Immobilization

The most common way to immobilize enzymes is the attachment to a suitable carrier by means of physical adsorption or covalent binding. Physical adsorption can rely on hydrophobic interactions, van der Waal's forces, or ionic interactions, and is therefore regarded as a gentle and reversible method [51, 56–58]. Carriers that possess negatively/positively charged ions at their surface (i.e., anion exchange resins, aminated cationic supports) or are hydrophobic (i.e., octadecyl methacrylate or poly‐acrylic resins) are suitable to immobilize POIs via ionic or hydrophobic interactions, respectively. The drawback of this approach is that it often suffers from the leakage of the enzyme from the support material [59].

Covalent binding, on the other hand, results in the irreversible attachment of the POI to the surface of the carrier and can be achieved by crosslinking suitable surface residues of the enzyme and the functionalized carrier [39, 51, 57, 58]. Primary amines (N‐terminus and the ε‐amino group of lysines) and the thiol groups of cysteines are commonly employed for covalent binding. For instance, the N‐terminus of the POI can be reacted with aldehyde groups at the surface of the support, which are then converted to stable secondary amines by the addition of reducing agents (single‐point covalent immobilization), and lysine residues can form covalent bonds with glyoxyl supports under alkaline conditions (multipoint covalent immobilization). By modulating the residues involved in covalent binding, it is possible to minimize the loss of activity for the POI; however, covalent binding often induces unintended conformational changes to the active site of the enzyme, reducing the activity drastically [57, 58].

Affinity binding can likewise be used for immobilization, where the POI is fused with an affinity tag and attached to a carrier harboring a complementary affinity ligand. Such affinity tags can be attached to the POI genetically (i.e., His‐tag, Halo‐tag), or added post‐translationally to the POI in a covalent manner (e.g., biotin), where the POI will be immobilized to the carrier via the corresponding ligand (i.e., nitrilotriacetic acid [NTA] chelated Ni^2+^, Halo‐tag ligand, and streptavidin, respectively) in a highly specific and directed manner [39, 60]. However, affinity binding can be costly, due to the high costs of the carrier material, and in the cases where the affinity tag is attached genetically, the approach might need further optimizations so as to not compromise the activity of the enzyme [57].

Various polymers (i.e., sol–gels, hydrogels) or solid matrices (i.e., porous ceramic, activated carbon) can be employed to entrap or encapsulate POIs, where the POI remains unbound but surrounded by the semi‐permeable matrix formed by the polymeric support [57, 61]. This is often achieved by mixing the isolated enzyme directly with the polymer solution or the porous support. The method is widely applicable but can suffer from leakage and mass transfer limitations [51].

Likewise, carrier‐dependent immobilization methods can be employed in a combinatorial manner, where for instance, heterofunctional carrier materials (i.e., glyoxyl‐agarose, amino‐epoxy carriers) can facilitate the initial adsorption of the POI followed by covalent binding, or the POI can be adsorbed to suitable porous polymers, which then entrap it [38, 39].

### 2.2 Carrier‐Independent, Ex Vivo Methods for Enzyme Immobilization

CLEAs and their predecessor, CLECs, represent carrier‐free immobilization methods, which rely on a cross‐linking agent [62]. In the case of CLECs, the POI is purified upon release from the cells, batch crystallized, and further cross‐linked via glutaraldehyde. The CLECs method yields stable enzyme crystals with tailorable sizes making them suitable for a wide range of applications, however, due to the labor intensiveness of the method combined with high costs, they were later replaced by CLEAs.

CLEAs are produced by precipitating the POI without denaturing it (i.e., by salting out at their optimal pH and low temperature), followed by cross‐linking, whereby the reactive ε‐amino groups of lysines from neighboring POIs are permanently linked [39, 41]. While various di‐aldehydes can be used for this purpose, glutaraldehyde is the most commonly employed cross‐linking agent. Notably, different POIs can be cross‐linked with the approach, for instance, multi‐CLEAs and combi‐CLEAs contain multiple enzymes for noncascade and cascade reactions, respectively. Furthermore, CLEAs can be combined with carrier materials, such as silica, or even magnetic particles (the latter giving rise to mCLEAs, see Section 4.1.1), by adding the carriers to the enzyme precipitate during the cross‐linking step. These hybrid immobilizates can be further tailored and have been employed for various applications as reviewed by Sheldon [19], as well as Costa from the perspective of biodiesel production [63].

### 2.3 Carrier‐Independent, In Vivo Methods for Enzyme Immobilization

In the recent years, the fully in vivo immobilization of enzymes has gained popularity. In vivo immobilization is achieved by producing the POI in insoluble (or phase‐separated) manner in living cells [9, 52–55]. More specifically, the gene encoding the POI is either fused to that encoding an inclusion body (IB)–inducing tag (catalytically‐active inclusion bodies [CatIBs]), an insoluble biopolymer (polyhydroxyalkanoate [PHA] granules), a viral capsid (virus‐like particles, VLPs), an insoluble protein crystal (Cry3aa crystals), an intrinsically disordered protein domain (liquid–liquid phase separation [LLPS]), or the POI is post‐translationally displayed onto such insoluble proteinaceous particles using protein interaction motifs (i.e., SpyTag/SpyCatcher, E‐/K‐ coil, SYNZIP1/SYNZIP2 coiled‐coil systems [64, 65, 66]).

IBs are often produced in bacteria as a consequence of strong overexpression of recombinant genes, for example, in *E. coli*. IBs, which mainly consist of aggregated proteins and are typically associated with a lack of activity, have a size of 50–800 nm and are dense and water‐insoluble particles [53]. As such, they can be easily recovered after cell lysis via centrifugation, and have been used as scaffolds where functional proteins have been displayed onto. This is commonly achieved by inducing IB formation via the overproduction of aggregation‐prone proteins (i.e., polyhydroxybutyrate [PHB] synthase, cellulose binding domains [CBDs] [54, 67, 68]), which are fused to a bait tag (i.e., negatively charged α‐helical E‐coil, which can form a coiled‐coil with its lysine‐rich pair (K‐coil), or the use of leucine zipper bait/prey domains). Such IBs with bait domains are in turn used to “catch” POIs fused to prey domains, which results in the post‐translational “attachment” of the POI to the insoluble IB particle, which can then be isolated.

CatIBs [9, 52, 69, 70] represent a special form of IBs which retain their catalytic activity. They are typically produced by the genetic fusion of an aggregation‐prone peptide/domain to the gene encoding the POI, which is then expressed under conditions which favor IB formation, as well as the retention of catalytic activity (low temperature, high induction strength). CatIBs, similar to IBs, can be recovered easily, and the POI incorporated into CatIBs typically retains its activity to a certain extent, depending on the enzyme and aggregation‐inducing tag employed (i.e., 1%–58% of the activity of the soluble, purified enzyme [71, 72, 73]). Wide applicability, ease of production, high protein content of the immobilizates, and increased stability have made the CatIB method a popular choice for enzyme immobilization in the recent years. Exemplary cases of magnetized CatIBs also exist, which are described in Section 4.1.1.

PHA granules are biopolymers that are produced by various bacteria and some archaea as natural energy storage units. They consist of a hydrophobic core surrounded by a proteinaceous layer with a size ranging from 100 to 500 nm [9, 74]. PHA synthesis depends on the following three enzymes: acetyl‐CoA acetyltransferase (PhaA), acetoacetyl‐CoA reductase (PhaB), and PHA synthase (PhaC), where PhaC remains covalently attached to the surface of the PHA granules upon synthesis. Therefore, PhaC, which as an isolated protein can be used for IB display approaches as outlined above, can also serve as an anchor to display immobilization targets to the surface of the PHA granules. In addition to PhaC, certain phasins such as PhaF/PhaP were also shown to localize to PHA granules and can, therefore, be utilized for POI display [9], which is achieved by fusing the POI encoding gene to the gene encoding PhaC/PhaF/PhaP directly, or fusing various bait–prey tags [75, 76], with the fusion proteins being then overproduced in organisms capable of PHA synthesis.

Cry3Aa is a crystal‐forming protein from *Bacillus thuringiensis*, with toxicity towards certain insects. Besides their well‐known pesticidal use, Cry3Aa crystals, which can be up to 1 µm in size, can also be used for POI entrapment [9]. The POI and Cry3Aa can be co‐produced to enable entrapment of the target without the need for genetic fusion [77], however, genetic fusions of *cry3aa* and the gene encoding the POI can also covalently attach the POI inside of the Cry3Aa crystals [78, 79].

VLPs (size between 20 and 200 nm) are made up of viral capsid proteins (CPs) capable of self‐assembly, which can be used to entrap or display POIs [80, 81]. For encapsulation, the gene of an immobilization target is either fused to that encoding a viral CP, which can then self‐assemble and thereby entrap the POI (e.g., Cowpea chlorotic mottle virus capsid), or, the gene encoding the POI is fused to those encoding scaffolding proteins which are involved in capsid assembly and allow entrapment of the POI within the interior of the capsid (i.e., *Salmonella* virus P22 [82, 83, 84, 85]). In addition to encapsulation or entrapment by VLPs, POIs can also be displayed onto the surface of the capsids, which can be accomplished by engineering the CPs to accommodate post‐translational attachment of POIs, which results in a VLP decorated with the POI [9, 81, 86]. Similar to other approaches, E‐/K‐coil and SpyTag/SpyCatcher pairs have been commonly combined with the VLP‐based immobilization approach [84, 87, 88], and magnetized VLPs also exist (see Section 4.1.2)

Finally, LLPS can serve as an immobilization method, where oligomerization, macromolecular crowding effects, and often the presence of intrinsically disordered proteins/regions result in the formation of a dilute phase, which spontaneously demixes from the solution, forming a protein condensate as an isolatable phase [9, 89, 90]. Certain proteins such as DDX3 RNA helicase and spider silk protein spidroin 1 contain intrinsically disordered regions (RGG domain and I16, respectively [91, 92]), which can be fused in tandem to trigger LLPS, and can then be used to direct POIs to the protein condensates via bait–prey protein interaction motifs, or direct fusion of the gene encoding the POI to such intrinsically disordered proteins. Intrinsically disordered proteins or domains can be further fused to certain photoreceptors, which oligomerize upon illumination, such as cryptochromes (Cry2/Cry2Olig) [93] and blue‐light using flavin (BLUF) proteins (e.g., PixE/D) [94] to enable light‐dependent LLPS.

Due to their versatility, magnetic materials, containing, for example, iron, nickel, and cobalt that respond to a magnetic field, are widely used in various industries. Applications have long been of technical nature (smart devices, data storage, sensing applications) [95] but extend to biomedicine (drug delivery, imaging) [96] and biotechnology (enzyme immobilization, waste water treatment) [28, 97]. For the understanding of the properties of magnetic materials, it is instrumental to understand the fundamental concepts that describe and categorize magnetic materials. Therefore, a very brief excursion into the physical principles of magnetism is needed.

### 2.4 Magnetic Properties Relevant for Enzyme Immobilization

**FIGURE 2:**
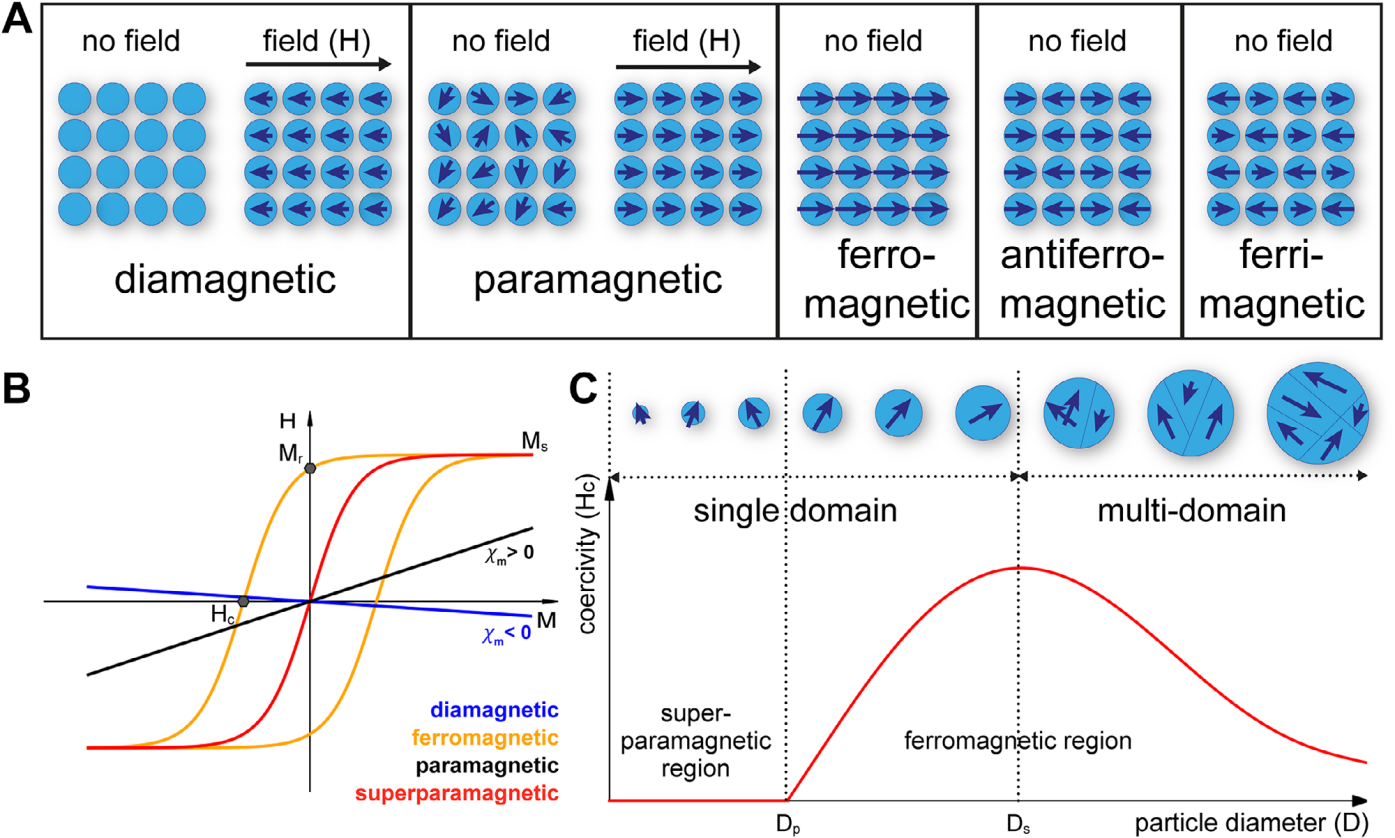
Properties of magnetic materials. Schematic representation of the microscopic structures of diamagnetic, paramagnetic, and ferromagnetic, antiferromagnetic and ferrimagnetic materials, both at rest and under the influence of a magnetic field H (A). Magnetization curves (*M*–*H*) of diamagnetic, paramagnetic, ferromagnetic, paramagnetic, and superparamagnetic materials (B). *M_s_*: saturation magnetization, corresponding to the maximum magnetic moment per unit volume of a given magnetic material; *M_r_*: remanent magnetization, which corresponds to the remaining magnetization of a material once the applied is removed; *H_c_*: coercivity, which is a measure for the reverse field needed to drive the magnetization to zero after *M_s_* was reached. $\chi_{m}:$ magnetic susceptibility ($\chi_{m}$ = *M*/*H*) describes how a magnetic system interacts with an external magnetic field; diamagnetic and paramagnetic materials show a small negative and larger positive magnetic susceptibility ($\chi_{m}$), respectively. Ferromagnetic materials show a large saturation magnetization (Mr) and a remanent magnetization and coercivity once the applied field is removed. Superparamagnetic particles show no remanent magnetization and a coercivity of zero once the magnetic field is removed (red line). Relationship between coercivity (*H_c_*) of a magnetic particle and its diameter (C). As particle size decreases, domain walls in multidomain particles disappear, causing *H_c_* to increase until the particle size reaches *D_s_*. Further reduction leads to thermal energy overcoming magnetic anisotropy, reducing *H_c_* and resulting in superparamagnetism when the particle size falls below *D_p_*. See Saini et al. [98] for an excellent introduction to the topic.

The phenomenon of magnetism at the atomic level can be attributed to a large extent to the motion and spin of electrons (as charged particles) within atoms. Firstly, electrons possess an intrinsic quantum property called spin (representing the intrinsic angular momentum of the electron, even though the electron is not actually spinning), creating a magnetic moment, so each electron behaves like a tiny magnet. Secondly, electrons orbit the nucleus, and this movement generates a magnetic field, with the combined effect of these orbits contributing to the atom's overall magnetic moment. Materials exhibit magnetism when many atoms coherently align their magnetic moments to create a macroscopic magnetic field (Figure 2A). In contrast to electron‐induced magnetism, nuclear magnetism, which arises from the magnetic moments of atomic nuclei, plays a much subtler role. Each nucleus with an odd number of protons or neutrons has a net magnetic moment due to the spins of these nucleons. Since nuclear magnetic moments are significantly weaker than those of electrons (because the magnetic dipole moment of charged elementary particles like electrons and protons is inversely proportional to their mass, and the mass of a proton is 1837 times that of an electron), we will focus on different forms of magnetism that arise from moving electrons [98].

The magnetic properties of a certain material are determined by several intrinsic properties. The saturation magnetization (*M_s_*) represents the maximum magnetization that a material can achieve per unit volume when subjected to an external magnetic field. The magnetization curve (*M*–*H* curve) (Figure 2B) of the material shows the relationship between the magnetic field strength (*H*) and the magnetization (*M*) in the material. Magnetic susceptibility ($\chi_{m})$ essentially describes how a magnetic system interacts with an external magnetic field. In addition, the remanent magnetization (*M_r_*), which is the magnetization that remains in a magnetic material after an external magnetic field has been removed, and the coercivity (*H_c_*) (Figure 2C), that is the intensity of the external field required to reduce the magnetization of the material to zero after it has been magnetized to saturation, are important parameters.

The six fundamental types of magnetism that can be distinguished based on *M*, *M_r_*, $\;\chi_{m}$, *H_c_*, and the *M*–*H* curve of the material are diamagnetism, paramagnetism, ferromagnetism, antiferromagnetism, ferrimagnetism, and superparamagnetism (Figure 2) [98]. Since these properties are important for the application of the corresponding magnetic materials, we will here provide a brief overview (see, e.g., Saini et al. [98] for details).

For diamagnetic materials, the magnetic moment is directly proportional to the strength of the external magnetic field, and the induced magnetic field is oriented opposite to the applied field (Figure 2A), which results in a small, negative magnetic susceptibility (Figure 2B) to external magnetic fields. These materials have no unpaired electrons, and their magnetic properties arise from slight asymmetric changes in the orbital motion of electrons in response to an external magnetic field. Examples of diamagnetic materials include inert gases, most organic molecules, and salts like NaCl [98].

Paramagnetic materials exhibit a positive magnetic susceptibility to external magnetic fields (Figure 2B). Their magnetic susceptibility is larger as compared to diamagnetic materials and the magnetic moments of the atoms or molecules orient with the applied magnetic field, with the paramagnetic moment being proportional to the strength of the external magnetic field (Figure 2A). In absence of an external magnetic field, the internal magnetic field of paramagnetic substances will be net zero, making them nonmagnetic (Figure 2A). Paramagnetic materials typically have unpaired electrons that contribute to their magnetic properties. Examples of paramagnetic materials include aluminum, platinum, titanium, and certain metal complexes and ions such as those of iron (Fe^3+^), manganese (Mn^2+^), and gadolinium (Gd^3+^) [98].

Ferromagenetic materials have a very high magnetic susceptibility (Figure 2B) and maintain their magnetization even when the applied magnetic field is removed (Figure 2A,B). In contrast to diamagnetism and paramagnetism, which are properties of atoms, ions, or individual molecules, ferromagnetism arises from the collective behavior of groups of atoms or molecules within solid crystals. Unmagnetized ferromagnetic materials contain domains with random direction of magnetization that cancel each other out. When placed into a magnetic field, the magnetic moments of the individual domains align parallel to the external magnetic field. Thus, essentially, ferromagnetism derives from an orderly arrangement of paramagnetic dipole moments. Ferromagnetic materials include permanent magnets made of, for example, iron, nickel, and cobalt or magnetite (Fe_3_O_4_) [98].

Similar to ferromagnetic and antiferromagnetic materials, ferrimagnetic materials are magnetically ordered substances where magnetic moments are arranged in an orderly manner. Ferrimagnetic substances show a lower magnetic susceptibility than ferromagnetic substances. Such materials have both parallel and antiparallel ordering of their constituent paramagnetic moments and because of excess parallel over antiparallel ordering ferrimagnetic substances display net positive magnetization (Figure 2A). Multidomain ferri‐ and ferromagnetic materials will achieve saturation magnetization even in weak external fields [98]. The most widely known ferrimagnetic substance is ferrite, a ceramic compound composed of mostly iron oxide (Fe_2_O_3_) and other elements like zinc or barium [99]. In antiferromagnetic materials, neighboring paramagnetic dipole moments align antiparallel to each other, resulting in a net zero magnetization (Figure 2A). Magnetite (Fe_3_O_4_) and greigite (Fe_3_S_4_), the sulfur equivalent of magnetite, are the only known ferro‐ or ferrimagnetic minerals found in living cells. For instance, magnetotactic bacteria encapsulate magnetite within a lipid‐bilayer membrane, forming structures known as magnetosomes [100] (see Section 4.2.1).

Superparamagnetism is a form of magnetism that occurs in nanometer‐sized ferromagnetic or ferrimagnetic materials (Figure 2C). When the size of these particles is sufficiently small (e.g., <35 nm for Fe_3_O_4_), they can exhibit magnetic behavior similar to paramagnetism despite being made of materials that are typically ferromagnetic or ferrimagnetic. When exposed to an external magnetic field, a sample containing many of these particles will magnetize similarly to multidomain materials, achieving saturation magnetization even in weak external fields (Figure 2B). However, once the external field is removed, Brownian relaxation (i.e., the physical rotation of the particles due to ambient thermal fluctuations [101]) and other processes will cause the magnetic dipole moments of individual superparamagnetic particles to become randomly oriented, resulting in no net magnetization retained by the material [98] (Figure 2B). Due to their small size, superparamagnetic particles are used widely in biological imaging applications, for example, as contrast agent in magnetic resonance imaging (MRI) [102].

## 3 Conventional Magnetic (Nano)Carrier Materials for Enzyme Immobilization

### 3.1 General Properties of Conventional Magnetic Carrier Materials

In the biocatalysis/biotechnology field, magnetic carrier materials of different sizes, ranging from magnetic nanoparticles (MNPs) to larger MBs (also known as magnetic particles or magnetic microspheres), have been widely used for enzymes immobilization [21, 28, 103–105]. Similarly, magnetic particles come in different shapes, ranging from the most widely used MNPs and MBs with spherical morphology to anisotropic nanoparticles like cubes, rods, disks, flowers but also more complex architectures like hollow spheres, stars, tetrapods or nanoplatelets, and so forth [106, 107]. While particles with a smaller size, due to their larger surface‐to‐volume ratio, provide more binding sites for enzyme immobilization and hence enable improved loading, larger particles are easier to recover magnetically. In addition, the shape of the employed nanomaterials can significantly influence enzymatic activity. Nanotubes and other nanostructures, due to their smaller size, exhibit larger radii of curvature, thus helping to reduce undesirable protein‐to‐protein interactions by allowing greater center‐to‐center distances between adjacent immobilized enzyme molecules [108]. In conclusion, both size and shape are instrumental for efficient enzyme immobilization and magnetic recovery to enable efficient immobilizate recycling [21]. Furthermore, as with all immobilization methods, the loading density and the mode of attachment to the support (e.g., covalent attachment vs. adsorption/affinity binding) and hence the associated differences in binding strength, durability, and reusability of the magnetic immobilizate play an important role in determining the efficiency of the immobilization method. This is not only true for the in the following presented MNP/MB immobilizates, but likewise extends to in vivo‐produced magnetic enzyme immobilizates (see Chapter 4), where these parameters are even more difficult to control. Another important issue for enabling efficient enzyme immobilization are surface functionalizations of the magnetic particles, which can significantly influence enzyme affinity, enzyme loading, the stability of tmagnetic enzyme immobilizates, as well as the leaching propensity (i.e., the loss of enzyme due to improper fixation), dispersibility, solubility, and biocompatibility of the particles [109]. In conclusion, optimization of these properties can provide better carrier materials for application in biomedicine and biocatalysis alike.

Since there are excellent recent reviews already covering these topics (see, e.g., Melo et al. [104], Holyavka et al. [110], Cavalcante et al. [21], Papatola et al. [27], Comanescu [109]), and the focus of this review is on biologically produced magnetic enzyme immobilizates, we will only provide a very brief (by no means complete) overview, which focusses on the recent developments.

### 3.2 Magnetic Beads (MBs) for the Immobilization of Enzymes

**FIGURE 3:**
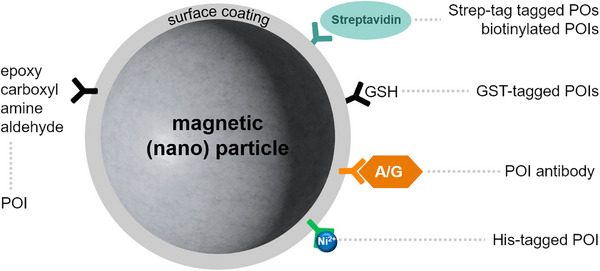
Architecture of magnetic beads (MB) and magnetic nanoparticles (MNPs) used for the immobilization of enzymes. MBs and MNPs usually consist of a ferro‐ or ferrimagnetic core (usually magnetite, but also other iron oxides like hematite and maghemite, spinel ferrites, etc.) often coated with nontoxic polymer coatings (chitosan, polyvinyl alcohol, polyethylene glycol, etc.) [27, 113] and functionalized with a number of ligands that enable POI binding. Those include amongst others, as nitrilotriacetic acid (NTA) chelated Ni^2+^ (binding His‐tagged POIs) [115], glutathione (GSH) (binding glutathione‐S‐transferase [GST] tagged POIs (see, e.g., [116]), protein A/G (allowing POI immunoprecipitation via POI specific antibodies) (see, e.g., [114, 118]), and streptavidin (binding biotinylated proteins or proteins with attached Strep‐tag) (see, e.g., [117]). Traditionally, POIs were attached covalently by crosslinking the POI with surface‐displayed functional groups such as, for instance, epoxy, carboxyl, amine, or aldehyde groups [119].

Nowadays, the most simple and easy method for immobilizing enzymes on magnetic carrier materials is the bioaffinity‐based immobilization on commercial MBs. For such MB systems, affinity binding generally relies on the use of an activated support (e.g., streptavidin, metal chelates bound as ligands to the surface of the MB) and short polypeptide tags or whole proteins fused to the POI that bind to the support‐displayed ligand. Commercially available MB systems, which are larger than MNPs (250 nm to 30 µm) [111, 112] usually consist of cross‐linked agarose encapsulating a ferro‐ or ferrimagnetic core (typically magnetite). In addition, other types of polymer coatings can be used, including amongst others, polyvinylalcohol, chitosan, and alginate [113] (Figure 3). Commercial MBs are available with a wide array of ligands such as NTA chelated Ni^2+^ (for His‐tagged proteins), glutathione (for glutathione‐S‐transferase‐tagged proteins), protein A/G for immunoprecipitation and streptavidin (for Strep‐tagged or biotinylated proteins) [114, 115, 116, 117, 118]. Alternatively, and traditionally more common, is the covalent immobilization of enzymes/proteins on (magnetic) solid supports [119], which is achieved by covalently linking functional groups coating the solid support (e.g., epoxy, carboxyl, amine, or aldehyde groups) and amino acid residues present on the surface of the enzyme [119].

For linking the enzyme to the support, a cross‐linking agent such as glutaraldehyde is needed, which, for example, targets the ε‐amino group of lysine residues [119]. In particular, superparamagnetic particles offer a number of benefits, including ease of separation and suitability for automation. MB systems have been utilized extensively for enzyme ligand/inhibitor screening/fishing approaches, for diagnostic biosensing applications, mostly in the biomedical field, for example, for the detection of bacteria, viruses, and other pathogens [120], as well as, to a more limited extent, in biocatalysis and biotechnology, for example, to enable mixing in microfluidic bioreactor systems [22, 121] or the setup of cascade reactions [122] including cofactor regeneration [123]. While such studies often employed macroscopic MBs, nanometer‐sized MNPs have also been used [21].

### 3.3 Magnetic Nanoparticles (MNPs) for Enzyme Immobilization

MNPs are biodegradable, biocompatible, cost‐effective, and can be customized by establishing specific surface chemistries to improve enzyme binding, specificity, and activity [27]. Their responsiveness to external magnetic fields enables easy and efficient biocatalyst separation, which simplifies purification steps, thereby reducing time and costs. Due to their size, MNPs offer a high surface‐to‐volume ratio, which enables enhanced enzyme loading and therefore superior catalytic efficiency compared to other enzyme carrier materials [27]. Iron oxides used for the synthesis of MNPs include Fe_3_O_4_ (magnetite), Fe_2_O_3_ (hematite), γ‐Fe_2_O_3_ (maghemite), and FeO [21]. Traditionally, spinel ferrite–based MNPs (MeFe_2_O_4_, Me = Fe*2* ^+^, Co^2+^, Mg^2+^, Mn^2+^, Zn^2+^, etc.) have been the most used materials for biocatalyst immobilization. Spinel ferrites are a class of magnetic materials with a crystal structure similar to that of the mineral spinel (MgAl_2_​O_4_​) [124]. Their unique structural characteristics and versatile crystal chemistry enable the tuning of their magnetic properties, opening interesting opportunities for a wide range of applications [124]. Although bare MNPs have proven to be good candidates for enzyme immobilization, biocatalyst performance can be affected by undirected immobilization of the enzyme, where active site structure or access is affected, resulting in lowered catalytic efficiency or mass transfer limitations [27, 125]. To address this issue, MNP surface modifications using biocompatible, natural molecules have been developed, including polymer coatings with nontoxic polymers like chitosan, polyvinyl alcohol, polyethylene glycol, and polyacrylic acid [27] (Figure 3). Most commonly, immobilization has been achieved by covalent attachment, cross‐linking, physical adsorption or co‐precipitation [27]. More recently, like for MBs (see Section 3.2), immobilization was also enabled by affinity binding of a His‐tagged POI to, for example, MNPs functionalized with Ni‐NTA functional groups [115] or NiFe_2_O_4_‐based MNPs [126, 127] (Figure 3). Over the last decades, MNPs have been widely used in biomedicine for MRI, targeted drug delivery, magnetic hyperthermia therapy, environmental remediation, and enzyme immobilization, where they, due to their unique physicochemical and magnetic properties, nowadays represent highly versatile immobilization carrier materials [21].

## 4 Biologically Produced Magnetic Carrier Materials for the Immobilization of Enzymes

In recent years, driven by human ingenuity and advances in synthetic biology and protein design, numerous solely biological, in vivo immobilization methods [9] have been developed. Most of the strategies rely on molecular biological methods that allow the display or encapsulation of a POI in/on biogenic materials. Generally, the following two principles are distinguished based on the nature of the separation of the immobilizate from the solute environment of the cell: (i) membrane‐free microcompartments such as carboxysomes [128], metabolosomes [129], bacterial PHA granules [130], VLPs [86] and (ii) membrane‐separated compartments such as liposomes [131], membrane vesicles [132], polymersomes [133], and magnetosomes [30, 134–137]. In addition to these naturally existing, architecturally complex compartments, also simpler, protein‐based materials exist. For instance, conventional, inactive bacterial IBs [67] on which a POI is displayed, catalytically‐active IBs (CatIBs) [52, 72, 73, 138] which incorporate correctly folded protein into a heterogeneous matrix of unfolded and partially folded POI, protein‐crystals, which encapsulate a POI (such as Cry3Aa protein–based crystals of *Bacillus thuringiensis* [77]) and plant forizymes have all been utilized for enzyme immobilization [139] (see Section 2.3).

In most instances, attachment of the POI is realized by molecular biological fusion [52, 72, 92, 140, 141] to specific anchor proteins that mediate incorporation into or display onto the surface of the “compartment” or protein matrix. In addition, post‐translational covalent attachment (e.g., via the SpyTag/SpyCatcher strategy [75, 142]) or via the use of specific protein–protein interactions (e.g., E/K coiled coils, leucine zippers [54, 67]) has also been reported. One general exception is the CatIB strategy, as it does not rely on such protein interaction domains, but instead utilizes the fusion of an aggregation‐prone protein domain or peptide to the POI to form the immobilizate.

In the following, we will focus mostly on strategies that have been used for the in vivo generation of magnetic enzyme aggregates. In some cases, mixed methods have been reported, where in vivo–produced enzyme immobilizates or compartments are subsequently magnetized ex vivo by chemical means.

### 4.1 Hybrid In Vivo/Ex Vivo Methods for the Generation of Magnetic Enzyme Immobilizates

#### 4.1.1 Magnetized CatIBs and CLEAs—Ex Vivo Prepared Magnetic Protein Aggregates

**FIGURE 4:**
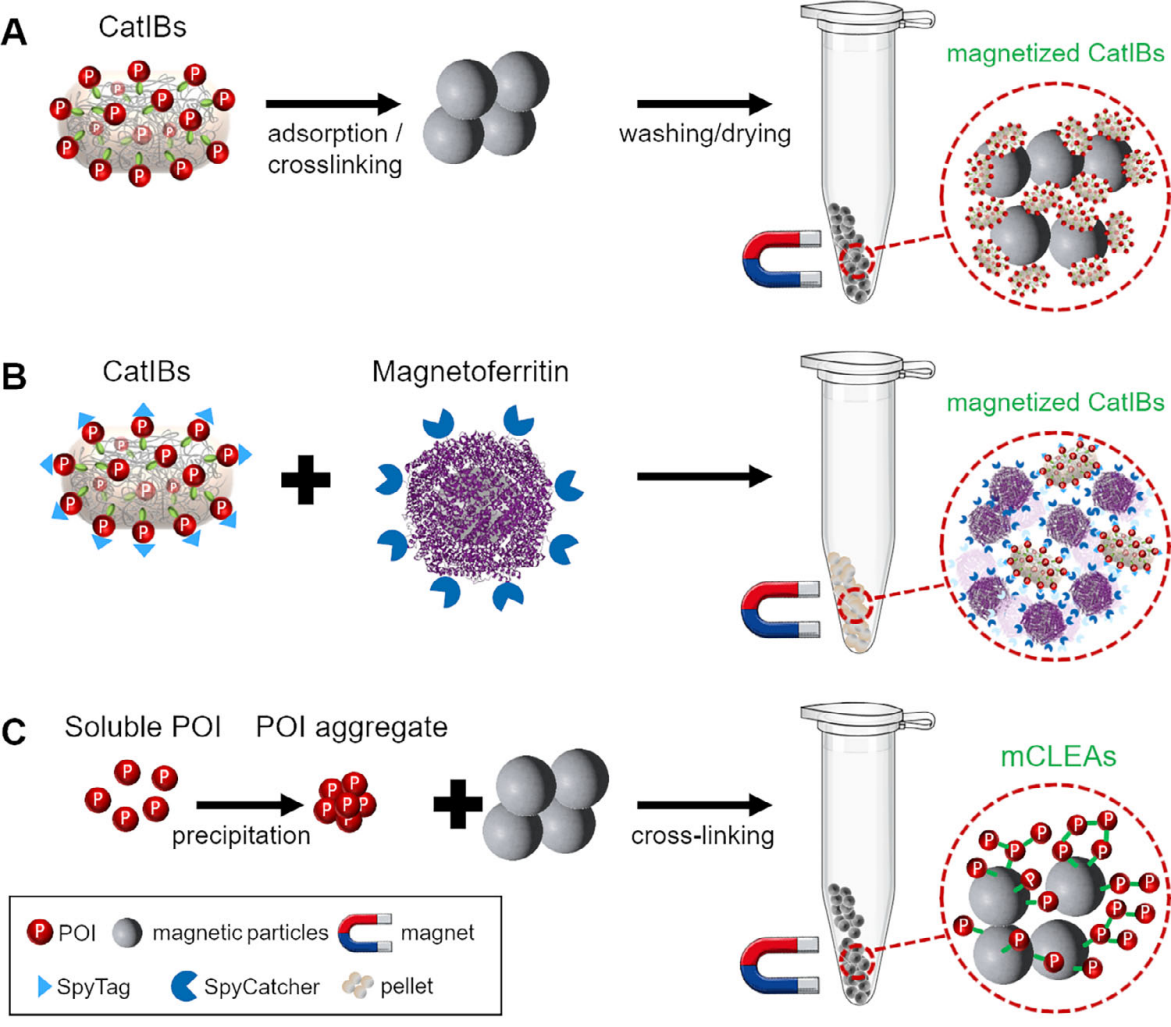
Strategies for preparing magnetized CatIBs and mCLEAs. CatIBs can be adsorbed onto/crosslinked to magnetic particles and can be subsequently washed and dried to obtain magnetic CatIBs [144] (A). Alternatively, CatIBs can be magnetized by utilizing ferritin with a magnetic core (magnetoferritin), where the POI and ferritin possess a complementary protein–protein interaction domain such as SpyTag and SpyCatcher, allowing magnetoferritin to irreversibly bind CatIBs [37] (B). mCLEAs can be prepared by the precipitation of soluble POIs, followed by mixing the resulting aggregates with magnetic particles and cross‐linking [146] (C).

As mentioned in Section 2.3, CatIBs represent in vivo*–*produced immobilizates generated by the fusion of an aggregation‐prone protein/peptide tag to a target enzyme, which leads to the formation of IBs consisting mainly of the target enzyme that retains its activity. Furthermore, CatIBs were also shown to be magnetizable, either via mixing with or cross‐linking to MNPs or very recently by the attachment to soluble ferritin cages via protein–protein interactions [37, 143, 144] (Figure 4A,B). For instance, magnetic CatIBs of the green fluorescent protein (GFP), a sialic acid aldolase, and an UDP‐glucose pyrophosphorylase were generated by simply mixing them with magnetic iron oxide particles, followed by washing and drying steps [143]. This was achieved either over several days in a freezer under slow drying, or rapidly via direct freezing or lyophilization, where the resulting CatIBs could be attracted to magnets. The magnetized CatIBs of sialic acid aldolase and UDP‐glucose pyrophosphorylase were of high stability and could be recycled up to 10 and 50 times, respectively. Further cross‐linkage by glutaraldehyde was also performed, where cross‐linking provided no added benefit to the magnetized CatIBs. In a different approach, we have recently attempted to magnetize CatIBs of an alcohol dehydrogenase [73] by post‐translational linkage to soluble ferritin cages of a magnetically improved *E. coli* ferritin variant [145] using the SpyTag/SpyCatcher interaction [37]. While the strategy produced CatIBs that could be purified with magnets with high efficiency, it involved the fusion of the large SpyCatcher peptide to the C‐terminus of the enzyme which compromised its activity, in line with previous observations for C‐terminal fusions of the employed alcohol dehydrogenase POI [73]. Since ferritin‐based CatIB magnetization requires the fusion of a CatIB‐inducing tag, as well as a protein–protein interaction motif to the target enzyme, its applicability may be limited to enzymes that tolerate both N‐ and C‐terminal fusions, or alternatively, the utilization of CatIB‐inducing tags that tolerate the direct fusion of such protein domains to the CatIB tag itself (i.e., where the CatIB formation efficiency would not be negatively impacted by the presence of an additional protein domain).

An alternative to magnetic CatIBs, magnetic cross‐linked enzyme aggregates (mCLEAs) were developed by combining CLEAs (see Section 2.2) with magnetic particles. Here, mCLEAs are produced by precipitating the enzymes from crude cell extracts, followed by cross‐linking in the presence of magnetic particles, typically magnetite (reviewed by Sheldon et al. [19]) (see Figure 4C). The magnetic particles are often amino‐functionalized using 3‐aminopropyl triethoxysilane [146] to facilitate cross‐linking via glutaraldehyde; however, the use of nonfunctionalized magnetic particles such as silica‐coated carbonyl iron is also reported, where the silica coating serves as a protective agent to prevent oxidation and dissolution of the carbonyl iron particles [147]. The magnetic particles used in the mCLEA method generally have a size between 1 and 15 µm, which can be used to separate the immobilizates easily under an applied magnetic field [19].

mCLEAs have been successfully produced for numerous industrially relevant enzymes up to date and have found application in bioremediation, biodiesel production, and the production of pharmaceuticals. For example, mCLEAs of horseradish peroxidase and glucose oxidase were used for the degradation of dyes [148], lipase‐mCLEAs were utilized for the production of fatty acid propyl esters from various oils [36], and penicillin G acylase mCLEAs were shown to have potential for the production of antibiotics [149]. Furthermore, the high stability (temperature and pH tolerance) and recyclability of mCLEAs have been widely demonstrated, where the cross‐linking conditions, choice of precipitating agent, and the employed magnetic particles were shown to have a large impact on the aforementioned parameters, which, therefore, have to be optimized per case [19, 148, 150–152].

In addition to the reaction cascade realized by the magnetic, combi‐CLEAs of horseradish peroxidase and glucose oxidase mentioned above (see Section 2.2), magnetic combi‐CLEAs of Pectinex Ultra Clear, which is a commercial multi‐enzyme preparation containing pectinase, cellulase, and hemicellulose activities, was used for biofilm inhibition [153]. Similarly, magnetic combi‐CLEAs of β‐galactosidase and l‐arabinose isomerase were used for the production of high‐value sugars [154]. Sequential utilization of differently functionalized mCLEAs has also been reported, as was shown for the production of the rare sugar d‐tagatose from lactose by β‐galactosidase and l‐arabinose isomerase mCLEAs in a step‐wise manner [155]. While both studies showed an improvement in productivity when compared to the utilization of free β‐galactosidase and l‐arabinose, sequential use of mCLEAs resulted in lower productivities when compared to the magnetic combi‐CLEA approach (13% vs. 25% conversion in 24 h), which might be attributed to the lack of substrate channeling effects in the case of the sequentially applied mCLEAs. Along the same lines, magnetic multi‐CLEAs of cellulase and hemicellulase, in combination with *Saccharomyces cerevisiae*, were used for the simultaneous saccharification and fermentation of pretreated wheat straw in noncascade reactions [156].

Furthermore, bioimprinting, which relies on inducing a conformational change to an enzyme via surfactants or crown ethers to promote enzymatic activity (i.e., changes to the secondary structure around the active site facilitating substrate access/lid opening mechanism of lipases [157, 158]), followed by “locking” of the active state by cross‐linking [159], has also been applied to mCLEAs. Here, imprinted polyphenol oxidase mCLEAs were prepared by the incubation of the enzyme with different imprinting agents, followed by cross‐linking onto functionalized iron‐oxide particles via glutaraldehyde [149]. The use of 4‐methyl catechol resulted in the highest activity improvement of the enzyme, where the resulting imprinted mCLEAs were used in the synthesis of l‐3,4‐dihydroxyphenylalanine (l‐dopa), a drug used in the treatment of various neurodegenerative diseases. Remarkably, the bioimprinted mCLEAs of polyphenol oxidase showed a 24‐fold increased l‐dopa yield, improved tolerance to high temperatures, as well as excellent process stability, evidenced by 95% activity retention after 10 reaction cycles.

Combination of the CatIB and mCLEAs methods was exemplified by the cross‐linking of MNPs together with CatIBs of the lipase B from *Candida antarctica* (CalB) [144]. Here, instead of precipitating the enzyme out of the solution, as is typical for the CLEA method, CalB CatIBs with a poly‐lysine tail were generated, where the tail facilitated cross‐linking. CalB CatIBs were isolated by centrifugation and cross‐linked by the addition of oxidized dextran. Additionally, wet CatIBs were mixed with Fe_3_O_4_ nanoparticles in the presence of oxidized dextran, yielding magnetic, cross‐linked CatIBs. The activities, as well as temperature and pH tolerance of the resulting CatIBs, cross‐linked CatIBs, and magnetic‐cross‐linked CatIBs were then compared, where the magnetic CatIBs retained 93% of their initial activity andshowed improved stability at high pH values and temperatures up to 70°C. Process stability of the magnetic CatIBs was also demonstrated, where they retained 85% of their initial activity after 10 reaction cycles.

Finally, a comparative study evaluating key parameters for enzyme immobilization in cascade reactions, using mCLEAs and enzymes immobilized on MNPs, revealed that mCLEAs retain higher activity and achieve greater productivity under optimal cross‐linker conditions. However, unlike MNPs, mCLEAs exhibited reduced magnetic strength after enzyme incorporation [160]. Here, a cascade reaction was realized by using two immobilized enzymes, namely (*R*)‐1‐phenylethanol dehydrogenase, and glucose dehydrogenase, for the production of (*R*)‐1‐(furan‐2‐yl) ethanol. The magnetic combi‐CLEAs displayed higher residual activity, stability, as well as improved tolerance to higher temperatures, demonstrating the viability of the mCLEA approach for cascade reactions [160].

#### 4.1.2 Virus‐Like Particles (VLPs) With Magnetic Core for Drug Delivery and Enzyme Immobilization?

**FIGURE 5:**
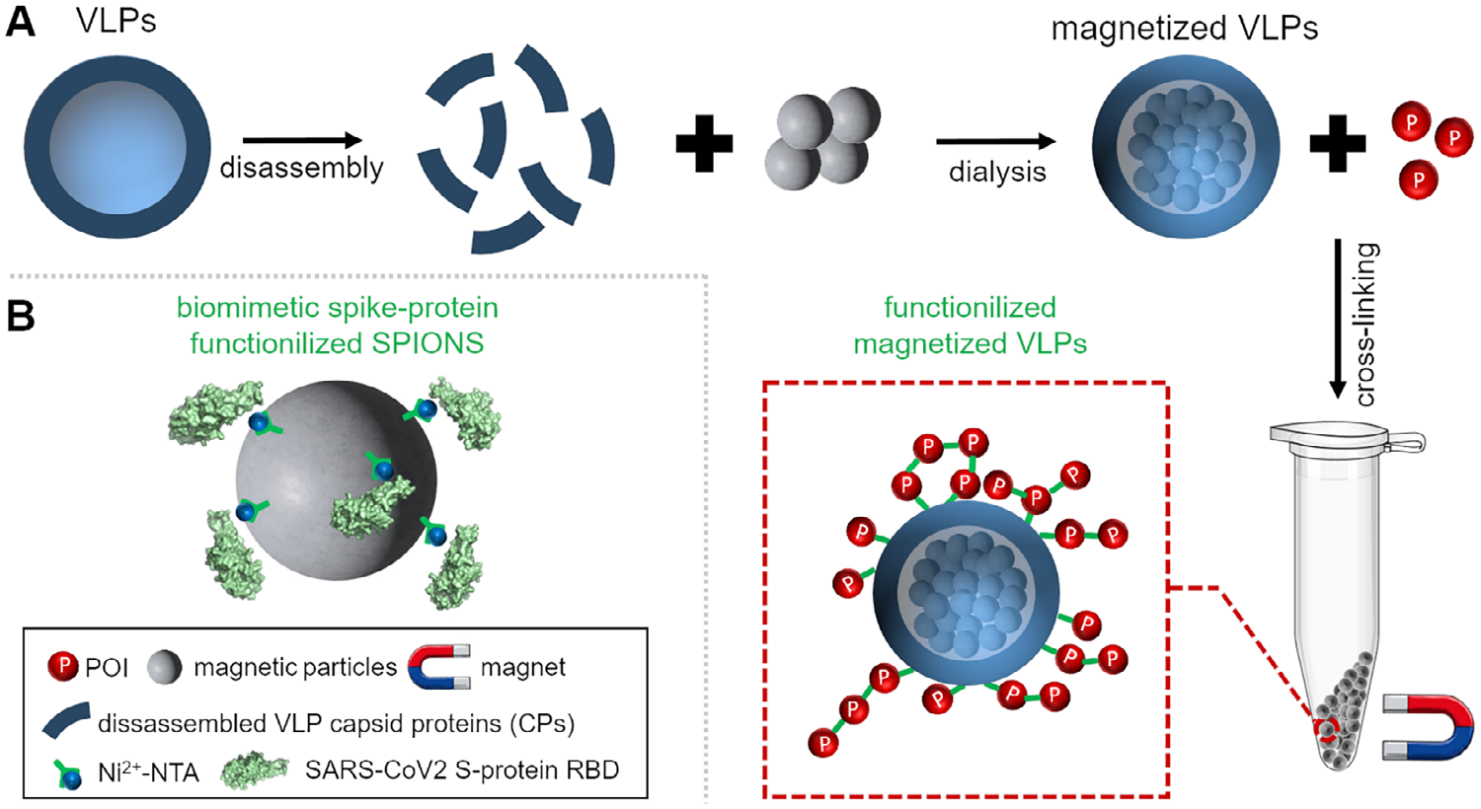
Strategies for preparing magnetized VLPs. VLPs are dissociated, mixed with functionalized MNPs and dialyzed against a suitable buffer, resulting in the self‐assembly of the CPs around the MNPs, yielding magnetized VLPs [163, 164], which subsequently can be cross‐linked with POIs (A). Alternatively, albeit likely not yielding native capsid‐like structures, biomimetic VLPs have been prepared by decorating functionalized superparamagnetic iron oxide nanoparticles (SPIONS) with His‐tagged SARS‐CoV2 spike(S) protein receptor‐binding domains (RBD) [165] (B). Structure of the RBD: PDB‐ID: 2AJF [253].

VLPs are self‐assembling structures made of viral proteins that closely resemble the native virus in both shape and size [9, 81]. Typically, VLPs consist of one or more viral capsid proteins (CPs), which can be readily synthesized in vivo through heterologous gene expression. Due to the lack of genetic material, they are noninfectious and do not replicate. Different CPs have been used for generating VLPs with different properties, and we and others have recently reviewed methods for POI encapsulation in and surface‐display on VLPs [9, 80, 81, 86, 161, 162]. Although not yet directly used for enzyme immobilization for biocatalytic purposes, it was shown in several studies that VLPs can be generated that contain a magnetic core [163, 164, 165] (Figure 5). In two studies, viral CPs, like the VP1 protein of the Simian virus 40 (SV40) [163] and the CPs of the brome mosaic virus (BMV) [164] were dissociated and mixed with functionalized MNPs. Functionalization, like in the case of the mentioned studies, coating of the MNPs with citrate in a two‐step ligand exchange reaction [163, 166] or functionalization with 1,2‐Distearoyl‐*sn*‐glycero‐3‐phosphoethanolamine‐N‐[carboxy‐(polyethylene glycol)_2000_](HOOC‐PEG‐PL) was needed to render the otherwise hydrophobic MNPs dispersible in aqueous media. When dialyzed against suitable buffers, the CPs self‐assembled into a proteinaceous MNP‐coating, yielding VLPs with magnetic core (Figure 5A). Magnetic VP1‐VLPs were then further functionalized by attaching the human epidermal growth factor (EGF) via a bifunctional crosslinking agent, which allowed the targeting of the corresponding EGF‐conjugated VP1‐VLPs to tumor cells ex vivo [163]. In another study, superparamagnetic iron oxide nanoparticles (SPIONS) were functionalized with N,N‐Bis(carboxymethyl)glycine (NTA) and its derivative, Nα,Nα‐Bis(carboxymethyl)‐L‐lysine (NTAL) to enable attachment of a His‐tagged version of the receptor binding domain (RBD) of the spike (S) protein derived from SARS‐CoV‐2, yielding biomimetic magnetic VLPs [165] (Figure 5B). The biomimetic SPION‐based VLPs, coated with the SARS‐CoV‐2 S protein RBD were used in proof‐of‐principle experiments as diagnostic tools to detect specific anti‐SARS‐CoV‐2 antibodies [165]. While not used explicitly for the immobilization of enzymes, VLPs with magnetic core represent an interesting extension of the widely used VLP‐immobilization strategy (see Section 2.3).

A comparison of the different hybrid in vivo/ex vivo methods for the generation of magnetic enzyme immobilizates, along with limitations, pros, and cons is presented in Table 1.

**TABLE 1:** Comparison of hybrid in vivo/ex vivo generated magnetic immobilizates.

Immobilization strategy	Engineering/Preparation effort	Production host	Magnetization (In Vivo/*ex vivo*)	Biocatalytic application^a^/Cascade use	Residual activity	Stability/Recycling of immobilizates	Pros and cons
Magnetized CatIBs	Low (toolbox available)	Bacteria *Escherichia coli*	Ex vivo/Mixing and or cross‐ linking to MNPs	Biocatalytic application/no	25–27 %CatIBs^b^	Good process stability/50 cycles, not reported [143]	Simple and cheap preparation/Low activity, no data on activity reduction due to immobilization (i.e., by comparison to a soluble purified enzyme)
Cross‐linked magnetized CatIBs	Low to medium (additional cross‐linking needed)	*E. coli*	Ex vivo/Mixing with MNPs	Biocatalytic application/no	93%^b^	Improved pH and thermostability/85% activity retention after 10 reaction cycles [144]	Simple and cheap preparation/No data on activity reduction due to immobilization
Magnetized CLEASs (mCLEAS)	Very low	*E. coli*	Ex vivo/Cross‐ linking to MNPs	Biocatalytic application/Yes (combi‐CLEAs)	13–135%^c^ [19]	Improved temperature, storage, and process stability/80–100% activity retention after six reaction cycles [19]	Simple preparation and recovery, widely used method/enzyme leaching at low pH, variable activities based on crosslinker or enzyme, no data on activity reduction due to immobilization
Bioimprited mCLEAs	Low to medium (bioimpriting using different agents needed)	*E. coli*	Ex vivo/Cross‐ linking to functionalized MNPs	Biocatalytic application/No	2350%^c^	Improved thermal and process stability/95% activity retention after 10 reaction cycles [149, 158]	High activity/not applicable to every enzyme, no data on activity reduction due to immobilization
Magnetized VLPs	Medium (depending on system and type of functionalization)	Plants, insect cells, mammalian cells [163, 164, 165]	Ex vivo/Mixing with functionalized MNPs	No/No	n.a.	n.a.	Production in eukaryotic hosts might enable native post‐translational modifications/No biocatalytic applications demonstrated

*Note:* See Chapter 4.1 for details about the strategies.

Abbreviations: n.a., not applicable; n.t., not tested.

^a^ Application categories include the following: (i) proof of concept, for example, demonstrating conversion of model substrates, (ii) biocatalytic application: optimized lab‐scale biocatalytic processes aimed at producing valuable compounds.

^b^ Compared to the activity of nonmagnetic CatIBs.

^c^ Compared to the activity of the soluble cell fraction, prior to magnetization. It should be noted that the actual residual activity of the immobilizates (i.e., the specific activity of the magnetic immobilizate, compared to the specific activity of the soluble, purified enzyme which is set to 100%) is not reported in the literature.

### 4.2 Magnetic Enzyme Immobilizates Solely Generated by In Vivo Methods

#### 4.2.1 Magnetosomes as, Tunable, Highly Versatile, Biologically Produced Enzyme Immobilizates

The discovery of magnetosomes as membrane‐separated compartments in magnetotactic bacteria is closely tied to the discovery of these microorganisms. Salvatore Bellini and Richard Blakemore independently discovered magnetotactic bacteria in the 1960s and 1970s, respectively [167]. Bellini first described them in freshwater samples in Padua, but his work, initially published in Italian only, was not widely disseminated until it was recently translated and published internationally [167, 168, 169]. In 1974, Blakemore found magnetotactic bacteria in Woods Hole sediment samples, and, using transmission electron microscopy and energy‐dispersive x‐ray microanalysis, demonstrated that these bacteria contained chains of iron‐rich magnetic inclusions [170], for which he later coined the term magnetosomes [171]. Magnetotactic bacteria do not form a phylogenetically coherent group, as they are found in various subgroups of the Proteobacteria phylum (α‐, β‐, and γ‐subgroups), the Nitrospirae phylum, and the PVC superphylum (Planctomycetes, Verrucomicrobia, and Chlamydiae) [172]. In particular, the α‐proteobacteria, *Magnetospirillum magneticum* AMB‐1 and *Magnetospirillum gryphiswaldense* MSR‐1, have been used extensively as model organisms to analyze the genetic and biochemical prerequisites of magnetosome formation and function.

**FIGURE 6:**
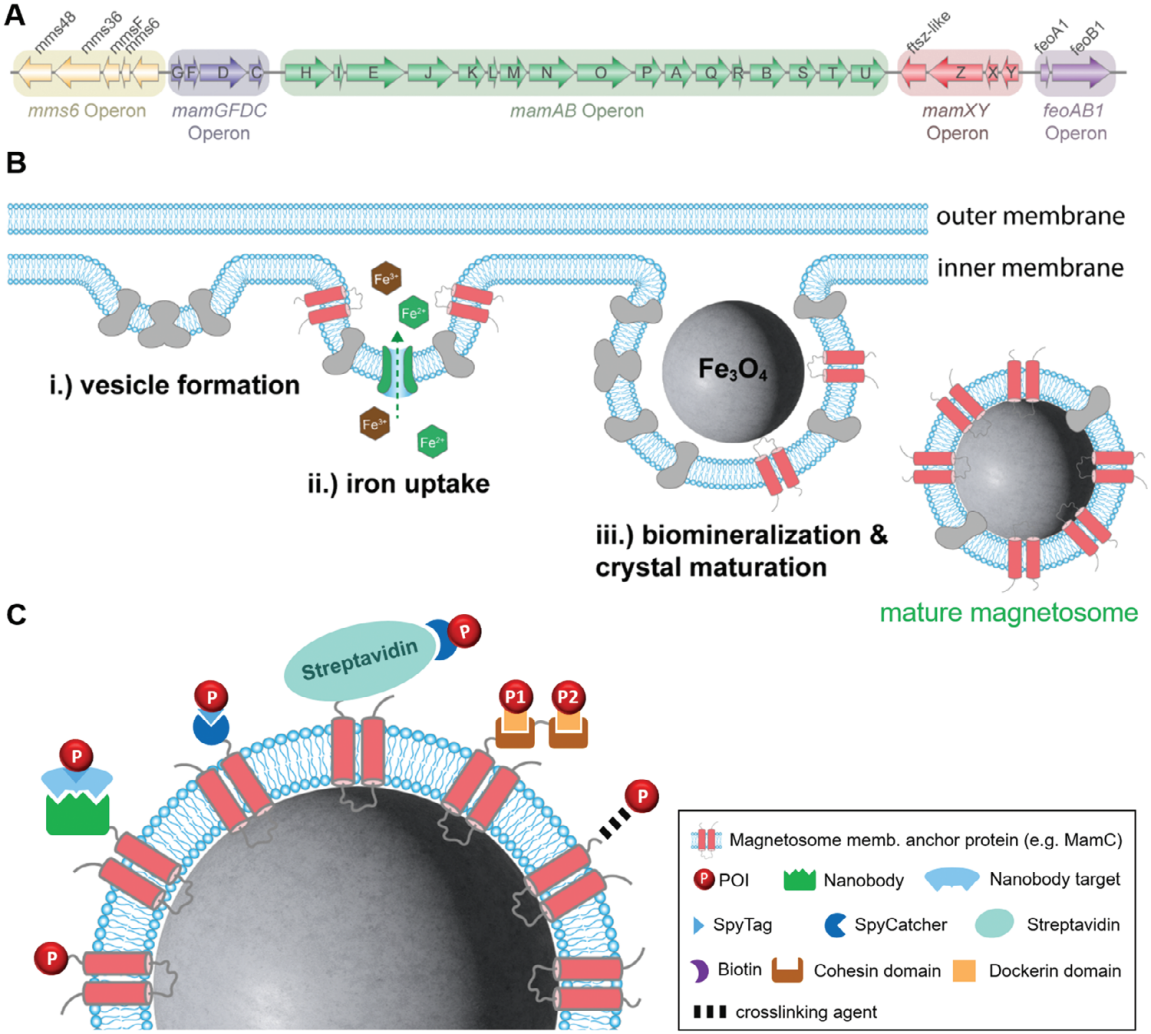
Magnetosome formation and strategies for the decoration of magnetosomes. In *M. gryphiswaldense*, all genes required for magnetosome biosynthesis are clustered in a genomic island, harboring >30 genes involved in magnetosome formation organized in six operons: *mmS6*, *mamGFDC*, *mamAB*, *mamX*Y, and *feoAB1* [134] (A). Magnetosome formation involves a number of key steps (B): (i) vesicle formation in the inner bacterial membrane, (ii) iron uptake, and (iii) biomineralization and magnetite crystal maturation. Subsequently, mature magnetosomes are assembled into chains aligned within the cytoplasm of the cells by actin‐like proteins (not shown) [134]. Several strategies have been presented for the decoration of magnetosomes with POIs (C), including direct fusion of the POI to magnetosome membrane proteins (MMPs) (see, e.g., [173]), glutaraldehyde‐mediated crosslinking [137], utilizing surface displayed by MMP‐nanobody fusions, with the POI being fused to the target of the respective nanobody (see, e.g., [178]), SpyTag/SpyCatcher interactions [30], Streptavidin/Biotin interactions [181]. In addition, multiple POIs have been displayed by the fusion of two orthogonal cohesin domains to an MMP, with the POIs being attached via their cognate dockerin domains (fused to the respective POI) [193].

In *M. gryphiswaldense*, all genes required for magnetosome biosynthesis are clustered in a genomic island, harboring >30 genes involved in magnetosome formation. Of these genes, the *mamAB* operon (Figure 6A highlighted in green) is essential and sufficient for magnetite biomineralization, with *mamE*, *mamL*, *mamM*, *mamO*, *mamQ*, and *mamB* being critical for magnetosome formation. In brief, magnetosome formation involves several key steps as follows: (i) oligomerization of magnetosome proteins (MamB, MamM, MamQ, MamL, recruiting MamI, MamE, and MamO) to initiate vesicle formation; (ii) invagination of the cytoplasmic membrane, iron transport into vesicle lumen by MamB, MamM, MamH, and MamZ; (iii) biomineralization involving nucleation (MamI), precipitation (likely MamO), and growth of magnetite crystals (multiple proteins including MamE) followed by the assembly of magnetosome chains (likely MamK, MamJ) [134] (Figure 6B). The mature magnetosomes contain membrane‐enclosed crystals of magnetite (Fe_3_O_4_) or greigite (Fe_3_S_4_) as their primary magnetic mineral components, which allow magnetotactic bacteria to orient themselves along geomagnetic fields for navigation. To enable in vivoimmobilization of POIs, magnetosomes can be functionalized by genetically fusing the POI‐encoding genes to the genes of highly abundant magnetosome membrane proteins (MMPs) like MamC, MamF, MamG, and the magnetosome‐surface‐associated protein MamA as membrane anchors (Table 2) (Figure 6C), often utilizing short linker polypeptides (e.g., Poly‐Glycine [(Gly_10_)] linker [173], flexible [(Gly‐Ser‐Thr)_17_] [30] linkers or α‐helical linker [LA(EAAAK)_4_AAA] [174, 175] linkers) as connectors spatially separating POI and the membrane anchor. To date, MamC (also called Mms13 *M. magneticum* AMB‐1) has been the most widely used magnetosome membrane anchor protein used for POI immobilization (Table 2). Expression of POI‐MMP fusions can either by achieved from multi‐copy replicative plasmids or by chromosomal integration of expression cassettes allowing both the high‐level constitutive or induced expression (see e.g., [176, 177]).

**TABLE 2:** Highly abundant magnetosome proteins used for immobilization of POIs.

Protein^a^/MW/copies per magnetosome^b^	Localization/Role	Fusion site^e^	POI		References
MamC^c^/12.4 kDa/100	Integral/Magnetite crystal growth	C‐terminal	Direct fusion strategy eGFP Glucuronidase Nanobodies RNase P Tropomyosin receptor kinase A Thyroid‐stimulating hormone receptor		[173, 178] [178, 180, 181] [179] [193] [194]
			Connector‐based strategy		
			*Connector*	*POI*	
			Cohesion/Dockerin	Endoglucanase β‐glucosidase	[193]
			SpyCatcher/SpyTag	eGFP Phenolic acid decarboxylase	[30]
MamF/12 kDa/21	Integral/Magnetite crystal growth	C‐terminal	eGFP Glucuronidase		[173]
MamG/8 kDa/6	Integral/Magnetite crystal growth	C‐terminal	eGFP Glucuronidase Glucose oxidase		[173]
MamA/24 kDa/17^d^	Surface/Magnetosome maturation	C‐terminal	eGFP Glucuronidase		[173, 175]

^a^ Protein names as described for *M. gryphiswaldense* MSR‐1 in [31].

^b^ Mms13 in *M. magneticum* AMB‐1.

^c^ Analyzed by semi‐quantitative mass spectrometry for [252]; copy numbers relative to mamC (100 copies per magnetosome [176]).

^d^ Since MamA is not an integral magnetosome membrane protein, relative abundance of 17% relative to MamC was assessed in [252], but no copy number value was provided.

^e^ Relative to magnetosome membrane protein.

Immobilized POIs vary widely in terms of complexity and function ranging from simple monomeric fluorescent reporters like the green fluorescent protein derivative eGFP [178] to multi‐subunit complexes, like the bacterial RNase P enzyme [179]. Additionally, multiple copies of the POI have been genetically fused to the membrane anchor resulting in magnetosomes decorated with a large number of enzyme copies and hence high activity as shown for magnetosomes functionalized with multiple copies of the glucoronidase GusA [31]. In addition to the direct fusion of the POI to the membrane anchor, modular connectors and nanobodies were fused and hence displayed on the magnetosome surface [180], which enables the recruitment of independently produced POIs to the magnetosome surface (Figure 6C). Here, in particular, the development of magnetosomes displaying nanobodies specific for GFP and mCherry [178, 180] is noteworthy, which moreover enables the binding of independently produced POIs labeled with GFP or mCherry, respectively [178]. Similarly, a bivalent nanobody targeting 3‐phenoxybenzoic acid (3‐PBA), a biomarker to detect exposure to pyrethroids, synthetic insecticides, implicated in long‐term health effects in humans, was biotinylated and immobilized onto streptavidin‐functionalized magnetosomes (Figure 6C), enabling 3‐PBA detection in urine [181]. Lately, also the SpyTag/SpyCatcher System [66] was used to recruit proteins to SpyCatcher decorated magnetosomes, with the SpyCatcher sequence being fused to *mamC* enabling recruitment and covalent attachment of SpyTag‐fused POIs [30] (Figure 6C).

The magnetic properties of magnetosomes are size‐dependent. Smaller particles, with a size of about 30 nm are superparamagnetic, meaning they do not retain a permanent magnetic moment at room temperature without an external magnetic field, reducing their tendency to agglomerate. Particles between 30 and 100 nm show stable single‐domain structure with remanent magnetization, while those over 100 nm become multidomain, exhibiting remanent, but reduced magnetization compared to single‐domain particles [182, 183]. Thus, the magnetic properties of magnetosomes vary within a narrow size range, allowing for the deliberate selection of superparamagnetic versus ferrimagnetic particles simply through size control [134]. In turn, size control can also be achieved by genetic manipulation, with different types of manipulation, like gene deletions or the integration of additional copies of magnetosome genes, yielding different magnetosome sizes [184, 185, 186, 187].

While in most studies, (functionalized) magnetosomes, for example, magnetic enzyme immobilizates, have been produced in their original hosts *M. gryphiswaldense* MSR‐1 or *M. magneticum* AMB‐1, recent studies have shown that magnetosome gene clusters can be transplanted to different hosts, for example, to the phototrophic model organism *Rhodospirillum rubrum* [188], photosynthetic *Rhodovastum atsumiense G2‐11* [189], and the nonmagnetotactic *Magnetospirillum* sp. 15‐1 [190]. Lately, the host range was increased further by testing 25 different α‐proteobacteria for their capability to heterologously produce magnetosomes [191]. Of the tested bacteria, *Cereibacter sphaeroides, Rhodoplanes elegans, Rhodopseudomonas pseudopalustris, Blastochloris viridis, Rhodoblastus acidophilus, Azospirillum brasilense*, and *Rhodomicrobium vannielii* produced magnetosome‐like particles, while A*grobacterium tumefaciens*, *Mesorhizobium japonicum, Rhodobacter capsulatus* SB1003, *Rhodobacter capsulatus* B10, and *Rubrivivax gelatinosus* failed to do so [191].

Since the first demonstration that magnetosomes can be used for enzyme immobilization in 1987, when glucose oxidase and uricase were immobilized on magnetosomes isolated from uncultured magnetotactic bacteria from a pond in Japan [136], applications of magnetosomes have diversified, now including the use as drug delivery vehicle, biosensing applications, bioremediation, as MRI contrast agent and of course enzyme immobilization [192]. In the following, we will provide a few examples for the use of magnetosomes‐based enzyme immobilizates, aiming to broadly cover the range of application fields rather than provide an exhaustive list.

It is not surprising that the applications of enzyme‐functionalized magnetosomes closely resemble those of traditional, chemically produced (functionalized) MNPs (see Section 3.3), extending from biomedicine (e.g., as biosensors/immunoassays, inhibitor screening approaches [181]), to bioremediation (e.g., enzymatic detoxification of organic pollutants [135]) and biocatalysis/biorefinery (e.g., biocatalytic synthesis of high‐value chemicals or biomass breakdown for biofuel production [30, 193]).

Studies on biomedical applications of protein‐functionalized magnetosomes have primarily focused on the display of medically relevant proteins, rather than enzymes, on the surface of magnetosomes. Those include amongst others, human tropomyosin receptor kinase A (TrkA) [193] and human thyroid‐stimulating hormone receptor (TSHR) [194]. TrkA, a receptor tyrosine kinase that plays a critical role in the development of the nervous system as the high‐affinity catalytic receptor of the neutrophin nerve growth factor (NGF), is an important target for drug screening because aberrant TrkA activity has been implicated in several diseases, including Alzheimer's disease, cancer, and depression [195, 196, 197]. TrkA was immobilized to magnetosomes by fusion to MamC and the functionalized magnetosomes were produced in *M. magneticum* AMB‐1. TrkA‐functionalize magnetosomes retained the NGF‐binding activity and tyrosine kinase activity [193]. Thus, the corresponding magnetosomes can be used for the functional analysis of TrkA as well as for high‐throughput drug discovery and screening. TSHR, a rhodopsin‐like GPCR, is crucial for thyroid hormone regulation [198]. Autoantibodies to TSHR (TRAbs) are linked to hyperthyroidism in Graves' disease, thus rendering the detection and quantification of TRAbs important for diagnosis. TSHR was immobilized to magnetosomes via fusion to MamC, and TSHR‐functionalized magnetosomes were produced in *M. magneticum* AMB‐1. The respective magnetosomes showed ligand (thyroid‐stimulating hormone, TSH) and autoantibody (M22 anti‐human TSHR autoantibody) binding capabilities, albeit with two orders of magnitude reduced ligand affinity compared to native human TSHR produced in a baculovirus expression system [199]. Interestingly, a recent study by the same authors showed that the affinity of TSHR‐functionalized magnetosomes can be improved by altering the magnetosome membrane composition via co‐expressing two phosphatidylcholine‐producing producing enzymes, namely phosphatidylcholine synthase and phosphatidylethanolamine N‐methyltransferase in *M. magneticum* AMB‐1 [200].

The capability of magnetotactic bacteria to absorb and immobilize other metals, like tellurium [201], cadmium [202], and selenium [203], along with their ability to be magnetically removed from sediment and water, renders them attractive for application in bioremediation, including waste water treatment [192]. Please note, however, that the foreign metals are not necessarily incorporated into magnetosomes, and the compound or metal to be removed can also negatively impact the magnetic properties of the bacteria [192]. In a similar manner, isolated magnetosomes functionalized with enzymes that enable, for example, the detoxification of organic pollutants can be used for bioremediation purposes. For this, Ginet and coworkers [135] fused the gene encoding an organophosphohydrolase (OPD) from *Flavobacterium* sp. ATCC 27551 to mamC and produced OPD‐functionalized magnetosomes in *M. magneticum* AMB‐1. OPD is known to degrade paraoxon [204], which is an active metabolite of the organophosphorus pesticide parathion [205]. By comparing the kinetics of purified OPD and OPD‐functionalized magnetosomes, the authors could show that the attachment to magnetosomes did not impact the affinity of the enzyme for its substrate nor its activity, and the magnetosome‐based immobilizate could be recycled multiple times without loss of activity [135].

To the best of our knowledge, biocatalytic applications of enzyme‐functionalized magnetosomes are rather rare, even though the first application, as briefly mentioned above, dates back to 1987 [136]. This is particularly true for fully in vivo–produced enzyme‐functionalized magnetosomes, where the enzyme‐encoding gene is genetically fused to a gene coding for a magnetosome membrane anchor protein, like MamC, enabling the in vivo decoration of the magnetosome surface with functional recombinant enzymes. Applications include the generation of a cellulase‐functionalized magnetosomes for cellulose hydrolysis [193] and the development of a magnetosome‐based platform for flow biocatalysis [30].

Cellulose represents the most abundant biomass source for bioethanol production. Therefore, immobilizing cellulases on solid supports can enhance handling, reusability, and stability, thereby improving space‐time yields of the corresponding biotransformation processes. To this end, Honda and coworkers [193] presented a connector‐based magnetosome immobilization system for the immobilization of the endoglucanase celCCA (EG) and the β‐glucosidase Ccel_2454 (BG), which collectively hydrolyze cellulose, in a cascade reaction, as demonstrated by the hydrolysis of the soluble cellulose, carboxymethyl cellulose, and the insoluble model cellulose Avicel [193]. The connector strategy, relied on the high‐affinity cohesin (Coh)—dockerin (Doc) interaction [206], which governs cellulosome assembly [207]. The authors fused two Coh domains, CohC of the scaffoldin CipA from *Clostridium thermocellum* and CohR of the scaffoldin ScaB from *Ruminococcus flavefaciens* in tandem to MamC, separated by a 10 amino acid linker peptide and produced CohC–CohR functionalized magnetosomes in *A. magneticum* AMB‐1 (Figure 6C). The two enzymes (without there signal sequence and native dockerin domain) were fused with the cognate Doc domains, DocC of the exoglucanase CelS from *C. thermocellum* and DocR of the scaffoldin‐like protein ScaA from *R. flavefaciens*. Assembly of the Coh‐functionalized magnetosomes with DocC‐EG and DocR‐BG yields a “synthetic cellulosome” in which EG breaks down the cellulose to oligosaccharides, and BG subsequently converts the oligosaccharides to glucose, with the close proximity of the two enzymes enabling efficient conversion [193]. By additionally attaching a CBD to MamC, resulting in a MamC–CohC–CohR–CBD fusion, the corresponding EG/BG‐CBD functionalized magnetosomes showed improved conversion of the insoluble model cellulose Avicel [193]. In a more recent study [30], another connector‐based system was utilized for immobilization of the dimeric phenolic acid decarboxylase (PAD) from *Enterbacter* sp., which is known for its capability to produce various styrene derivatives from phenolic compounds, offering a sustainable route to phenolic styrenes, which in turn can be used for the production of polymeric cross‐linkers for adhesion technologies [208]. As mentioned above, the strategy relied on the use of the SpyCatcher/SpyTag modules for covalent coupling of SpyTagged PAD to SpyCatcher‐functionalized magentosomes (SC‐magnetosomes), produced in *M. gryphiswaldense* MSR‐1 [134]. For functionalization of the magnetosomes, the SpyCatcher encoding sequences were fused to the 3′‐end of the MamC encoding gene, linked via a 17 amino acid Gly‐Ser‐Thr linker. In turn, the SpyTag was fused to the C‐terminus of PAD followed by a Hexahistidin (His_6_)‐tag (PAD‐ST‐His). ST and PAD modules were spatially separated by using a short GGGS‐linker as a connector. Incubation of purified SC‐magnetosomes with independently purified PAD‐ST‐His allowed covalent attachment of PAD‐ST‐His to SC‐magnetosomes. As controls, SC‐functionalized Dynabeads M270 with covalently attached PAD‐ST, or a fusion protein containing PAD and the HOB‐tag were used. The so‐called HOB‐tag, a variant of the Halo‐tag, which covalently binds to chlorohexyl suicide ligands [209], was attached to the beads using the streptavidin/biotin‐PEG‐chlorohexyl linker interaction for functionalization of streptavidin‐coated Dynabeads M280. The three bead systems were benchmarked against each other in a microfluidic magnetic flow reactor by following the conversion of *p*‐coumaric acid *to* p‐hydroxystyrene. Probably due to the smaller bead size of the magnetosome‐based immobilizates, superior activity per mg of carrier material was observed when compared to both Dynabead‐based PAD immobilizates. Using a continuous reaction mode, the magnetosome‐based PAD immobilizates also outperformed the other immobilizates with regard to operational stability, highlighting the stability of magnetosome‐based PAD immobilizates [30].

#### 4.2.2 MagR—A Controversial Magnetoreceptor Protein Used for the Generation of Magnetic Protein Immobilizates

**FIGURE 7:**
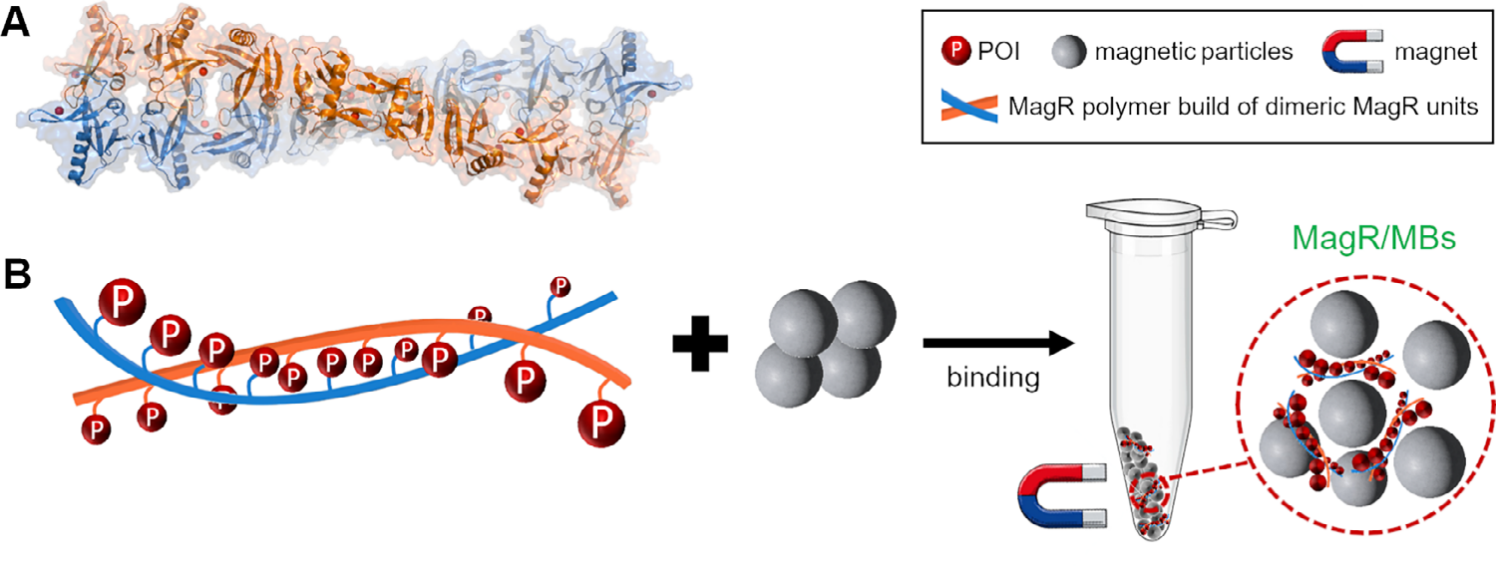
The putative magnetoreceptor protein MagR as magnetic scaffold for the immobilization of enzymes. MagR likely forms a multimeric rod‐like structure, as depicted here for the homologous IscA1 protein of *E. coli* (PDB‐ID: 1R94 [254]) (A). MagR's rod‐like structure was inferred from the analysis of crystal packing of the IscA1 structure and electron microscopy data for cIMagR [210]. Immobilization of enzymes was achieved by molecular biological fusion of a given POI to the C‐terminus of MagR. Direct fusion, without a linker, but also utilization of rigid and flexible linker polypeptides was tested [32]. Since the MagR C‐terminus protrudes from the proposed rod‐like polymer, POI fusion would result in the decoration of the MagR polymer with POI molecules (B, left part of the panel). Mixing crude cell lysates of MagR‐POI‐producing cells with Fe_3_O_4_–SiO_2_ magnetic beads allows for the magnetic purification and immobilization of the POI (B, right part of the panel).

In 2016, an Fe–S‐cluster assembly protein IscA1, renamed MagR by the authors, was identified in *Drosophila melanogaster* and found to be generally conserved in animals [210]. Based on biochemical and biophysical experiments, the authors suggested that MagR might represent the long sought‐after magnetoreceptor that, alone or together with cryptochrome (Cry) photoreceptors, another candidate magnetoreceptor protein, could facilitate the detection of the earth's magnetic field in animals [210]. It was suggested that the small (ca. 14 kDa) MagR protein forms a ferrimagnetic multimeric rod‐like structure (Figure 7A), which forms a complex with Cry that responds to magnetic fields in virtro. Magnetic properties were suggested to derive from a [2Fe–2S] cluster found in each of the 20 MagR monomers that formed the complex [210]. The MagR protein of the respective host was found to be able to interact with one (but not all) of the hosts' cryptochromes, which was tested for six selected species [210]. The authors showed that the MagR protein and the MagR/Cry complex can be enriched from a complex mixture using silica‐coated magnetite beads [210], which was utilized in subsequent studies for the one‐step purification and immobilization of MagR‐POI fusions from *E. coli* cell lysates [32, 35] (Figure 7B). In all cases, the POI was fused to the C‐terminus of the MagR protein and either *Columbia livia* (clMagR) [32] or *Drosophila melanogaster* (dmMagR) [35, 211] was used as magnetic “pull‐down” domain. Further, GFP, a lipase, an α‐l‐arabinofuranosidase, a pullulanase [32] and a QS signal hydrolase [35] were magnetically purified using Fe_3_O_4_–SiO_2_ MBs, with the immobilized lipase showing excellent operational stability over 20 consecutive reaction cycles [32]. Interestingly, different fusion constructs were tested with GFP as a target, revealing that the addition of a rigid or flexible linker had little impact on magnetic purification and the fluorescence of the fusion protein [32]. While these results sound very promising, at least from an application point of view, that is, for the generation of magnetic enzyme immobilizates, the physical properties and the functionality of MagR as a magnetoreceptor have been widely debated [212, 213, 214]. Unlike initially suggested [215], MagR fusions failed to induce significant membrane channel activity in mammalian cells in a magnetogenetic perturbation approach [216], which was later attributed to poor plasmid design and low expression efficiency by the authors of the initial study [217]. Along the same lines and based on calculations, it was later suggested that the number of iron atoms in MagR proteins is too low to detect magnetic fields effectively [212]. Last but not least, and most importantly for application of MagR for the generation of magnetic enzyme immobilizates, in a recent study Pekarsky et al. were unable to replicate the magnetic purification of MagR [214]. MagR binding to MBs was not quantitative and many *E. coli* host cell proteins also absorbed nonspecifically to the Fe_3_O_4_–SiO_2_ beads, prompting the authors to suggest that MagR is rather bound via strong ionic interactions to the beads than via magnetism [214]. It should however be noted that the authors failed to produce clMagR in soluble form in *E. coli* [214], which was previously used for the immobilization of various enzymes [32], and instead based their work dmMagR only [214]. Irrespective of its controversially discussed role as a magnetoreceptor and as a tool for magnetogenetic perturbation, from a biotechnological application point of view with regard to the generation of magnetic enzyme immobilizates, the verdict is still out whether or not MagR homologs might be useable.

#### 4.2.3 Supramolecular Ferritin Assemblies/Ferritin Crystals as a Carrier

Ferritin is a ubiquitous, non‐heme iron storage protein found in almost all domains of life. Ferritins are spherical proteins with a hollow core, with an inner and outer diameter of 8 and 12 nm, respectively. The protein shell is made up of 24 α‐helical subunits and the core can store up to 4500 iron atoms as ferrihydrite (5Fe_2_O_3_.9H_2_O) crystals [218, 219, 220]. While the mammalian ferritins are hetero‐24mers consisting of a heavy chain involved in the oxidation of Fe (II) to Fe (III) and a light chain involved in the mineralization of iron, bacterial ferritins are homo 24mers. Besides serving as an iron reservoir, ferritin protects cells from oxidative damage by mineralizing free iron which would otherwise lead to the formation of harmful hydroxyl radicals via the Fenton reaction [218, 219, 220].

In addition to iron‐loaded ferritin (magnetoferritin) being superparamagnetic, ferritin cages possess several interesting properties such as high thermostability, the ability to self‐assemble in aqueous solutions, and high tolerance to surface modifications. This has led to ferritin being employed in various biotechnological applications in recent years, including but not limited to, serving as a contrast agent in MRI, a drug delivery platform, and a nanobioreactor for the production of quantum dots and various nanomaterials such as carbon nanotubes [221]. Despite the superparamagnetic properties of iron‐loaded ferritin, individual ferritin cages exhibit limited magnetic properties due to the rather small size of the iron‐containing cavity, as well as the properties of ferrihydrite mineralized inside the ferritin cages, which has approximately eight‐fold less magnetic susceptibility than magnetite [212]. However, ferritin cages can be chemically or genetically manipulated to exhibit increased magnetic properties [145, 222], form supramolecular assemblies [34, 223, 224], can be entrapped within larger protein crystals [33], or loaded with iron ex vivo to sequester ferrimagnetic magnetite or maghemite [225]. As such, supramolecular assemblies of ferritin have found uses in biotechnology as a biologically produced carrier, and there are exemplary cases of such ferritin‐based scaffolds functionalized with enzymatic activity.

Various synthetic, thermoresponsive polymers were mixed with the ferritin from *Pyrococcus furiosus* to produce reversible, supramolecular assemblies in aqueous solution in a temperature‐dependent manner [224]. This was achieved by complexing ferritin with linear, di‐, and tri‐block copolymers consisting of poly[2‐(dimethylamino)ethyl methacrylate] and poly[di(ethylene glycol) methyl ether methacrylate] chains, where the former is cationized to facilitate the binding of the polymer to ferritin, and the latter exhibits hydrophilicity at low temperatures but is hydrophobic at temperatures above 33°C. The size of the ferritin supramolecular assemblies could be tailored depending on the use of di‐ or tri‐block polymers, temperature, and salt concentration, resulting in assemblies with sizes ranging between 300 and 800 nm.

**FIGURE 8:**
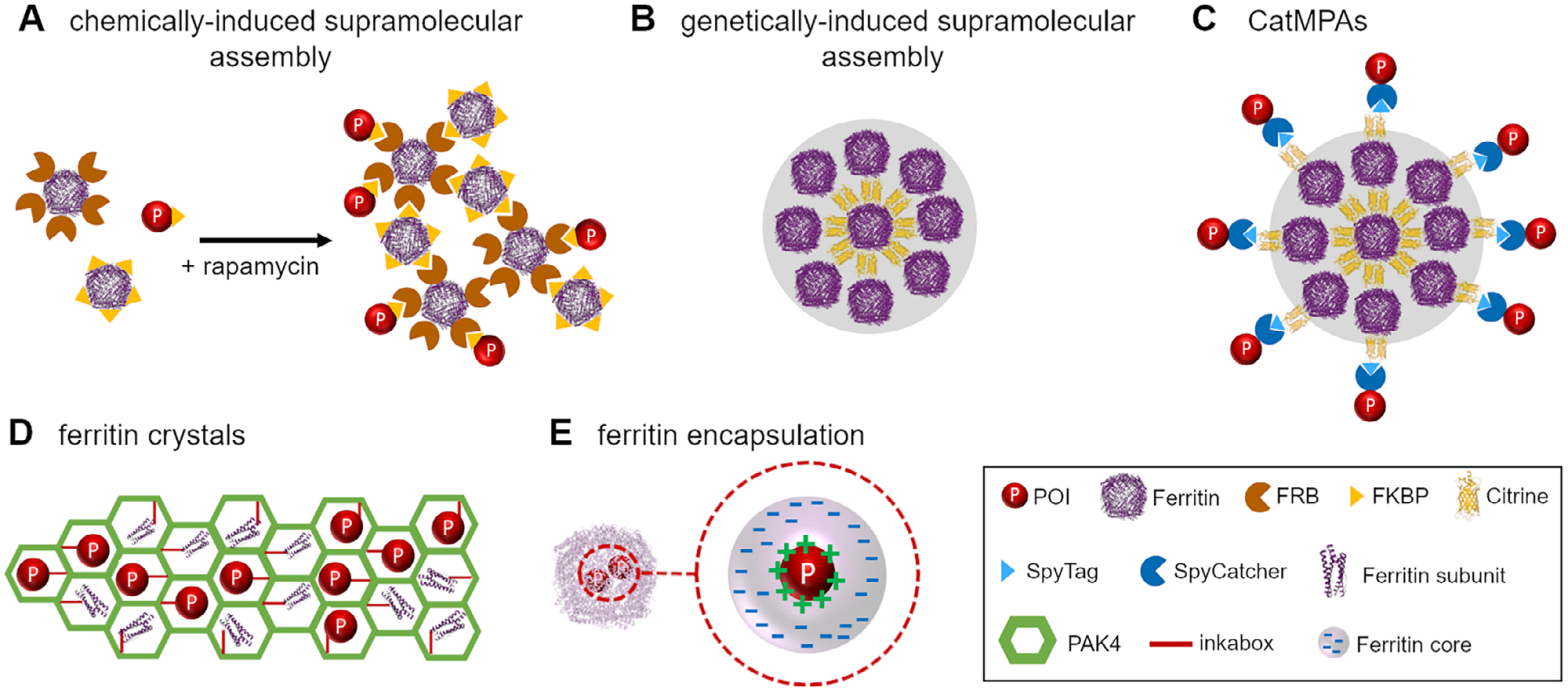
Various immobilization strategies utilizing ferritin. Chemically induced ferritin supramolecular assembly formation via three fusion proteins (A): fusion of FK506 binding protein (FKBP) to ferritin, fusion of rapamycin‐binding domain of mTOR (FRB) to ferritin, and the fusion of FKBP to the POI. Upon mixing all fusion proteins and induction by rapamycin, the depicted assembly is formed [34]. Genetically induced ferritin supramolecular assembly formation via the fusion of yellow fluorescent protein citrine to ferritin, where the assembly depends on the dimerization of citrines [223, 226] (B). Catalytically active magnetic protein aggregates (CatMPAs) formed by the fusion of citrine‐ferritin bearing a SpyTag, which allows the recruitment of a SpyCatcher bearing POI [37] (C). Ferritin crystals formed by the Cdc42 effector (PAK4) and the inkabox motif of PAK4 inhibitor protein Inka1 (inkabox) (D). Interaction of inkabox with PAK4 results in crystal formation, and fusions of ferritin‐inkabox‐PAK4, POI‐inkabox‐PAK4, and inkabox‐PAK4 can be coproduced to obtain ferritin and POI containing protein crystals [33]. Encapsulation by ferritin cages (E). Positively charged POI can be entrapped inside the negatively charged cavity of ferritin [231, 232, 234].

An example of genetic modification of ferritin for the supramolecular assembly is the fusion of a yellow fluorescent protein variant citrine to the heavy chain of human ferritin, where the citrine–ferritin fusion was shown to self‐assemble into clusters in HeLa cells, as well as in *E. coli* [223, 226] (see Figure 8B). Here, supramolecular assembly formation was postulated to be mainly driven by the dimerization of citrines, where each of the 24 subunits of ferritin fused to a citrine recruits another ferritin cage via the neighboring citrines, as evidenced by the lack of supramolecular ordering when the monomeric A206K mutant of citrine was fused to ferritin instead. Interestingly, the size, level of order, and organization of citrine–ferritin supramolecular assemblies seem to depend on the expression host. For instance, the assemblies in HeLa cells were shown to be of crystalline nature, with sizes ranging from 100 nm to more than 2 µm and showed different types of organizational structures [223], whereas the assemblies in *E. coli* were noncrystalline, present at only one cell pole, and were much smaller in comparison (≈500 nm) [223]. Furthermore, citrine–ferritin assemblies generated in *E. coli* could be obtained with ease upon cell lysis due to their self‐sedimenting nature, disrupted to individual citrine–ferritin monomers by the addition of chaotropic agents, and loaded with iron ex vivo [226].

Another example of a ferritin‐based supramolecular assembly relying on genetic fusions uses ferritin from *P. furiosus*, where the fusion of two different proteins which can form heterodimers in a chemically inducible manner, to ferritin was shown to form such assemblies (Figure 8A) [34]. Interestingly, the resulting ferritin assemblies could further be decorated with fluorescent reporter proteins, as well as the microtubule nucleating protein TPX2, to magnetically manipulate microtubules. This was achieved by generating individual fusions of the FK506 binding protein (FKBP) and rapamycin‐binding domain of mTOR (FRB) to ferritin, which were then purified and mixed ex vivo. Upon rapamycin supplementation, supramolecular ferritin assemblies with sizes ranging between 100 nm and a few micrometers could be observed, driven by the dimerization of FKBP and FRB fused to ferritin [34]. These assemblies could further be used to recruit GFP and mCherry, where additional fusions consisting of FRB‐GFP or FKBP‐mCherry were mixed with FKBP‐ferritin, or FRB‐ferritin, respectively [34]. The ferritins were then magnetized by ex vivo iron loading of the single FKBB/FRB‐ferritin cages, where magnetic manipulation of the ferritins was shown to be possible upon supramolecular assembly formation triggered by rapamycin, whereas individual cages did not respond to the magnetic field [34]. Finally, the iron‐loaded ferritin assemblies were decorated with the microtubule nucleating protein TPX2 and mCherry, induced via the mixtures of FKBP‐TPX2, FKBP‐mCherry, FRB‐ferritin, and FKBP‐ferritin fusions with rapamycin supplementation. The TPX2‐ferritin supramolecular assemblies could nucleate microtubule asters within *Xenopus* egg extracts, which also resulted in the entrapment of the TPX2‐ferritin assemblies within asters. The entrapment of the iron‐loaded TPX2‐ferritins in turn enabled the microtubule structure to respond to a magnetic field, where remarkably, the entire microtubule structure could be dragged under an induced magnetic field gradient. Furthermore, the presence of ferritin clusters decorated with TPX2 was shown to have an effect on the centering of the microtubule asters, which may be a promising discovery in terms of the manipulation of different cell processes [34].

As an alternative to ferritin‐based supramolecular assemblies, mutant ferritin subunits which can store iron individually [227, 228] can also be entrapped within biological crystals and magnetized by ex vivo iron‐loading, which was evidenced by the encapsulation of a ferritin in inka‐PAK4 crystals formed by the Cdc42 effector PAK4, and the inkabox motif of PAK4 inhibitor protein Inka1 [33] (see Figure 8D). Here, the strategy relies on the interaction of inkabox with PAK4 which leads to conformational changes leading to spontaneous crystallization of the complex, yielding inka‐PAK4 crystals. Inka‐PAK4 crystals can have cubical or spindle‐like morphologies, with sizes from a few microns to up to 200 µm. To entrap ferritin inside the inka‐PAK4 crystals, the gene encoding an engineered *P. furiosus* ferritin with improved iron accumulation properties was fused to that of PAK4 [33]. The resulting protein fusion (ftn‐PAK4) was then co‐produced together with inka‐PAK4 in mammalian cells, giving rise to ferritin‐containing crystals. While the ferritin crystals are postulated to contain more than 10 million ferritin subunits, they did not contain iron in vivo, and therefore had to be released from the cells and iron loaded ex vivo. Upon iron loading, ferritin crystals were shown to be pulled towards permanent magnets and did not influence each other upon the removal of the magnetic field, hinting at their paramagnetic properties [33]. Furthermore, the ferritin‐containing inka‐PAK4 crystals could be rendered fluorescent by the co‐production of the GFP‐PAK4 fusion protein together with inka‐PAK4 and ftn‐PAK4, demonstrating the approach to be principally compatible with POI recruitment [33].

In a similar vein, double‐chambered ferritin (where multivalent peptides and proteins are present at the surface of the ferritin nanocage) was used for targeted apoptosis of tumor cells without a cargo drug, which was accomplished by the fusion of a dual‐functional peptide, as well as GFP, to human ferritin [229]. Tumor targeting ability and cellular uptake of ferritin was conferred by the CGKRK peptide, which binds the p32 receptor overexpressed in cancer cells. Apoptosis on the other hand was triggered by the cytotoxic peptide (KLAKLAK)_2_, which disrupts the mitochondrial membrane [230]. Both peptides were fused N‐terminally to ferritin, whereas GFP was fused to the C‐terminus. Upon overproduction of the fusion protein in *E. coli* and subsequent purification, the dual‐functional peptide‐bearing ferritin was used to trigger apoptosis in vivoin xenograft mouse models.

Interestingly, ferritin was also used as a carrier to encapsulate enzymes, albeit with very limited capacity for entrapment (up to 3 enzymes/ferritin cage) due to the small size of the ferritin core when the size of target proteins is considered [231, 232] (Figure 8E). Ferritin from *Archaeoglobus fulgidus* has a unique, tetrahedrally symmetrical structure, and unlike the sub‐nanometer‐sized pores of conventional ferritins, possesses four, relatively large pores (4.5 nm, [233]). This property of the *A. fulgidus* ferritin was exploited to encapsulate a highly positively charged GFP variant (GFP+36) in the negatively charged interior cavity of ferritin, simply by mixing purified ferritin with GFP+36 [232]. The strategy was then transferred to encapsulate three enzymes, via the generation of GFP+36‐human carbonic anhydrase II, GFP+36‐retro‐aldolase, and GFP+36‐Kemp eliminase fusions, which resulted in the encapsulation of the enzymes within the ferritin cages driven by the positively charged GFP+36. Whether the cargo enzymes were taken up into pre‐assembled ferritin cages, or, if the cargo protein itself drives the self‐assembly of the ferritin cage was not clear, leaving the exact mechanism of cargo incorporation open to debate [232]. In a rather similar study, the same archaeal ferritin as well as its disassembly resistant mutant which lacked the large pores present in wild‐type protein were used to determine the mode of cargo uptake. Assembly of wild‐type ferritin around the GFP+36, as well as the incorporation of GFP+36 by the pre‐assembled, mutant ferritin, was demonstrated, indicating different means of cargo incorporation to be possible [234]. However, since the cargo protein is much larger than the pores of the mutant ferritin (4 nm) the study raises more questions about the exact mechanism of cargo incorporation by ferritin. It was postulated by the authors that the mechanism allowing large cargo uptake may function in a similar way to certain properties of the assembled viral capsid subunits, which were simulated to be capable of “coming on and off.” Furthermore, the same approach relying on *A. fulgidus* ferritin was tailored to encapsulate a genetically modified human carbonic anhydrase II without relying on GFP+36 [231]. Here, the POI contained 19 amino acid substitutions, which changed its net charge from −1 to +21. In this way, the mutant human carbonic anhydrase II could be encapsulated within ferritin cages without requiring GFP+36, however, the approach did not improve the amount of cargo that could be encapsulated (1–2 enzymes/ferritin cage). Nevertheless, purified ferritins containing human carbonic anhydrase II could be further immobilized covalently onto an agarose resin, where the enzyme activity was retained, and an improvement to its thermostability could be demonstrated [231].

Another example of enzyme immobilization using ferritin as a scaffold was accomplished via the decoration of ferritin cages with β‐glucosidase [235]. This was done via E‐coil bearing ferritin, and both K‐coil and His‐tag bearing β‐glucosidase, in a labor and purification‐intensive manner. In this case, both ferritin and β‐glucosidase were purified (by salting out from the supernatant of the cell lysate, followed by resuspension and size exclusion chromatography for the former, and affinity chromatography for the latter), dialyzed, and then further purified by size exclusion chromatography. The ferritin was then iron‐loaded ex vivo, followed by the purification of iron‐loaded ferritin via gel filtration, followed by a sucrose density gradient, and the decoration of iron‐loaded ferritins with β‐glucosidase by mixing the purified proteins. To retain the soluble iron‐loaded ferritin cage harboring the target enzyme, the proteins had to be further attached to a Ni‐NTA column via the His‐tag harboring β‐glucosidase. The ferritin‐β‐glucosidase mixture, which should normally not exhibit any magnetism due to its soluble nature, was described to be exposed to an undescribed “magnetic field,” where the recyclability was demonstrated via the retention of approximately half of the enzyme activity after four cycles [235].

Finally, we very recently described the generation of magnetic, ferritin‐based scaffolds called magnetic protein aggregates (MPAs), which could be further decorated with an alcohol dehydrogenase to yield catalytically active magnetic protein aggregates (CatMPAs, [37], Figure 8C). These MPAs were developed based on the fusion of citrine to a genetically engineered mutant of *E. coli* ferritin [145] with improved iron sequestering properties, similar to those first described by Bellapadrona et al. by the utilization of the human ferritin as described earlier in the text [223, 226]. However, unlike their human ferritin‐containing counterparts, MPAs containing the enhanced iron sequestering mutant ferritin displayed magnetic properties when produced in *E. coli* with iron supplementation, without the need for ex vivo iron loading. MPAs could either be isolated via centrifugation due to their insolubility, or purified with commercially available magnetic columns, albeit with 42% purification efficiency.

Furthermore, the CatMPA strategy was implemented as proof‐of‐concept for enzyme immobilization, where the SpyTag present at the N‐terminus of citrine‐mutant ferritin fusion and the SpyCatcher at the N‐terminus of an alcohol dehydrogenase enabled the recruitment of the soluble enzyme to the supramolecular assemblies of MPAs. The stability of the lyophilized CatMPAs was also demonstrated, where the alcohol dehydrogenase activity remained unchanged during a 5‐day incubation at room temperature, whereas the soluble, purified alcohol dehydrogenase lost up to 70% of its original activity in the same time period [37]. To provide a concise summary, Table 3 provides an overview of the various purely in vivo methods described above for generating magnetic enzyme immobilizates, highlighting their advantages, disadvantages, and limitations.

**TABLE 3:** Comparison of purely in vivo generated magnetic immobilizates.

Immobilization strategy	Engineering/Preparation effort	Production host	Magnetization (In Vivo/*ex vivo*)	Biocatalytic application^b^/Cascade use	Residual activity	Stability/Recycling of immobilizates^d^	Pros and cons
Magnetosomes^a^	Low to medium (only gene fusion and chromosomal integration or expression from plasmid possible)	Bacteria *Magnetospirillum magneticum* AMB‐1; *M. gryphiswaldense* MSR‐1; different α‐proteoacteria	In vivo	Proof of concept and biocatalytic application in flow microreactor/Yes	50%^c^, increased turnover of magnetosome immobilized enzyme compared to dynabead immobilized one [30]	65% activity retention after 96 h in continuous flow set up [30]	Superior magnetic properties, wide range of applications demonstrated/complex production, no production in *E. coli* demonstrated so far.
MagR	Low (only gene fusion to *magR* needed)	Bacteria *E. coli*	*In* In vivo + ex vivo binding to Fe_3_O_4_–SiO_2_ magnetic beads)	Proof of concept/No	120%–200%^d^ [32]	High, 70% activity retention after 20 reaction cycles [32]	Very simple system/Magnetic properties of isolated MagR protein under debate
Rapamycin‐induced assembly of FKBP/FRB‐fused ferritin	Medium (multiple constructs producing ferritin‐FKBP/FRB and the POI, need for chemical inducers)	*E. coli*	*Ex vivo*	Proof of concept/No	n.a.	n.a./n.a. [34]	Allows triggering of localized cellular responses/Multiple constructs and chemical inducers needed
Ferritin encapsulated in inka‐PAK4 crystals	Low to medium (multiple constructs producing ferritin, inka‐PAK4, and the POI is needed)	Mammalian cells	Ex vivo	No/No	n.a.	n.a./n.a. [33]	Larger crystals can be beneficial for POI recruitment/Not yet applied for enzyme immobilization
Decoration of ferritin cages	Medium (needs intense purification steps)	*E. coli*	Ex vivo	Proof of concept/No	100%^c^	No change to temperature or pH tolerance/56% after three reaction cycles [235]	Minimal activity loss/POI purification needed, POI must accommodate fusions to both termini, nonmagnetic properties of soluble ferritin cages
CatMPAs	Low (toolbox available)	*E. coli*	Ex vivo attachment to ferritin	Proof of concept/No	n.d.	n.t./n.t.	No need for carrier materials or additional immobilization steps/No data on activity reduction due to immobilization

*Note:* See Chapter 4.2 for details about the strategies.

Abbreviations: n.a., not applicable; n.t., not tested.

^a^ Only magnetosome applications aimed at biocatalytic use were considered in the here presented comparative analysis.

^b^ Application categories include the following: (i) proof of concept, for example, demonstrating the conversion of model substrates, (ii) biocatalytic/bioremediation application: optimized lab‐scale biocatalytic processes aimed at producing valuable compounds or use for bioremediation purposes.

^c^ Compared to TON of free enzyme.

^d^ Similar or exceeding the activity of free enzymes. Activity of clMagR‐enzymes purified by iron beads, compared to free enzymes purified by Ni‐NTA affinity chromatography.

## 5 Magnetizing Biotech—Future Research Avenues and Applications of Magnetic Enzyme Immobilizates

In vivo and ex vivo generated magnetic enzyme immobilizates hold great promise as enzyme carriers due to their ease of magnetic separation, which enables efficient recovery from reaction mixtures, large surface area, and hence high mass transfer efficiency [21, 236–238]. The latter is particularly advantageous for nanometer‐sized materials, including chemically synthesized MNPs (see Section 3.3), VLPs with magnetic core (Section 4.1.2), magnetosomes (Section 4.2.1), and ferritin‐based magnetic immobilizates such as ferritin‐clusters and ferritin‐crystals (Section 4.2.3).

While magnetosome‐based immobilizates are very promising from an application point of view, their production is currently still limited to magnetotactic bacteria or related α‐proteobacteria [191]. Moreover, the production of magnetosomes at a scale suitable for commercial applications remains challenging, since the cultivation of magnetotactic bacteria (e.g., *M. gryphiswaldense* MSR‐1, *M. magneticum* AMB‐1) is difficult due to their diverse metabolisms. As such, strictly controlled cultivation conditions (dissolved oxygen concentration, pH, nutrient composition, and salt concentrations) are needed to optimize yields [239]. For widespread industrial adoption however, the possibility to produce them in industrial hosts like *E. coli*, *Corynebacterium glutamicum*, *Bacillus subtilis* or yeasts such as *Saccharomyces cerevisiae* or *Pichia pastoris* would be highly desirable.

The use of ferritin‐based scaffolds like ferritin‐clusters, ferritin‐crystals, enzymes incorporated into ferritin cages and ferritin‐based MPAs is still in its infancy and is restricted by the limited magnetic properties of individual ferritin cages, which, however, can be increased by aggregation/clustering (see Section 4.2.3). Here, in particular, larger supramolecular assemblies such as the above‐described MPAs and their enzyme‐decorated counterparts with catalytic activity (CatMPAs, see Section 4.2.3), which can be produced in *E. coli* in iron‐loaded form, represent an interesting alternative to magnetosomes as biologically produced magnetic enzyme carrier materials.

Alternatively, in vivo–produced enzyme immobilizates could be obtained by utilizing a combination of different, already existing techniques. For example, VLPs with magnetic coregenerated by encapsulating or displaying ferritins, could potentially be obtained by co‐expression and assembly of VLPs and ferritins, with attachment of the ferritin to the in vivo–assembled VLP particle being driven by protein‐protein interaction motifs like the E‐coil/K‐coil or the SpyTag/SpyCatcher system. In addition to alternative methods for the production of magnetic enzyme immobilizates, future applications are poised to revolutionize various fields by leveraging advanced technologies and genetic engineering. Stimulus‐responsive enzymes and enzyme nano‐particles, which can be activated by local heating induced through high‐frequency magnetic fields, might offer precise control over biochemical processes [23, 240]. These developments also encompass the new field of magnetogenetics [241, 242], where magnetomechanical [243, 244] and magnetothermal [245] approaches are explored for precisely controlling cellular events, though controversies remain regarding their efficacy [246, 247, 248]. Additionally, genetically encoded MRI contrast agents based on magnetosomes [249], ferritin [250], or other proteins [251] are being developed, which can be targeted by functionalization of the magnetosome surface to specific cells (e.g., cancer cells) [249].

In conclusion, the advancement and application of in vivoand ex vivo–generated magnetic enzyme immobilizates underlines the transformative potential of integrating magnetic technologies into biocatalysis, biotechnology, and biomedicine. As challenges in scaling production and optimizing properties persist, this rapidly evolving field might offer promising solutions driving innovation, and expanding opportunities for efficient, precise, and targeted enzyme applications across various industries, thereby “magnetizing biotechnology”.

## Conflicts of Interest

The authors declare no conflicts of interest.

## Data Availability

Data sharing is not applicable to this article as no datasets were generated or analyzed during the current study.
